# The promoter architectural landscape of the *Salmonella* PhoP regulon

**DOI:** 10.1111/j.1365-2958.2012.08036.x

**Published:** 2012-03-27

**Authors:** Igor Zwir, Tammy Latifi, J Christian Perez, Henry Huang, Eduardo A Groisman

**Affiliations:** 1 Section of Microbial Pathogenesis, Yale School of Medicine295 Congress Avenue, 354D, New Haven, CT 06536, USA; 2 Department of Computer Science and Artificial Intelligence, University of GranadaE-18071 Granada, Spain; 3 Department of Molecular Microbiology, Washington University School of MedicineCampus Box 8230, 660 S. Euclid Ave., St. Louis, MO 63110, USA; 4 Howard Hughes Medical InstitutePO Box 27389, West Haven, CT 06516, USA; 5 Yale Microbial Diversity InstitutePO Box 27389, West Haven, CT 06516, USA

## Abstract

The DNA-binding protein PhoP controls virulence and Mg^2+^ homeostasis in the Gram-negative pathogen *Salmonella enterica* serovar Typhimurium. PhoP regulates expression of a large number of genes that differ both in their ancestry and in the biochemical functions and physiological roles of the encoded products. This suggests that PhoP-regulated genes are differentially expressed. To understand how a bacterial activator might generate varied gene expression behaviour, we investigated the *cis-*acting promoter features (i.e. the number of PhoP binding sites, as well as their orientation and location with respect to the sites bound by RNA polymerase and the sequences that constitute the PhoP binding sites) in 23 PhoP-activated promoters. Our results show that natural PhoP-activated promoters utilize only a limited number of combinations of *cis*-acting features – or promoter architectures. We determine that PhoP activates transcription by different mechanisms, and that ancestral and horizontally acquired PhoP-activated genes have distinct promoter architectures.

## Introduction

Bacterial activators stimulate gene transcription by binding to specific DNA sequences on target promoters where they make contact with RNA polymerase (RNAP) and/or alter the local DNA structure (Barnard *et al*., 2004). Activators may bind to a single or to multiple sites at a given promoter, in both possible orientations and at various distances from the site bound by RNAP (Li *et al*., 1997; Roy *et al*., 2004). Activator binding to a promoter may require cofactors that increase the sensitivity of the regulation (Pedersen *et al*., 1991; Browning and Busby, 2004) and/or overcome the silencing effects of nucleoid-associated proteins that predominantly affect genes that were horizontally acquired (Dorman, 2007). Although we understand at atomic resolution levels the interactions that certain transcriptional activators establish with their target sequences (Blanco *et al*., 2002; Browning and Busby, 2004; Mayo *et al*., 2006), it is still unclear which arrangement(s) of *cis*-acting regulatory features, such as the number, orientation, location and sequence of binding sites, can be utilized by a given activator to promote gene transcription. The particular arrangement of these features, which we refer to as promoter architecture, is of special interest for activators that control multiple targets. This is because genes co-regulated by a particular activator protein are often expressed in distinct fashions, and this could be due to the corresponding promoters having different architectures. Here we analyse the promoter architectures of genes directly activated by PhoP, a transcriptional activator that governs virulence and Mg^2+^ homeostasis in several enteric species (Groisman, 2001).

The PhoP protein regulates expression of ∼ 5% of the genes in the Gram-negative pathogen *Salmonella enterica* serovar Typhimurium (Harari *et al*., 2010). It carries out this task both directly, by binding to the corresponding promoter regions (Kato *et al*., 2003; Lejona *et al*., 2003; Shi *et al*., 2004; Bijlsma and Groisman, 2005; Shin and Groisman, 2005; Aguirre *et al*., 2006; Tu *et al*., 2006; Perez *et al*., 2008), and indirectly, by altering the levels and/or activity of regulatory proteins (Kato and Groisman, 2004; 2008; Tu *et al*., 2006) and RNAs (Moon and Gottesman, 2009; Lee and Groisman, 2010). Analysis of the genes directly activated by the PhoP protein has revealed that the majority exhibit a limited phylogenetic distribution and appear to have been acquired by horizontal gene transfer (Perez *et al*., 2009). Moreover, the encoded products differ in their biochemical functions and physiological roles (Groisman, 2001) (Table 1). This raises the possibility that they are produced in distinct amounts when *Salmonella* experiences PhoP-inducing conditions.

**Table 1:** Promoter data set corresponding to 23 PhoP-activated promoters

									PhoP box – RNAP distances		
										
Gene name	Function of encoded gene product	A	PhoP box sequence	#	O	A/R	Submotif	Score/submotif	−10	+1	Length of 5′ leader
*yrbL*	Putative cytoplasmic protein	I	TCGTTTAGGTTTTGTTTAA	1	D	A	S1	0.90	11	27	32
*phoP*	Response regulator	I	TGGTTTATTAACTGTTTAT	1	D	A	S1	0.88	12	33	34
*mgtA*	Mg^2+^ transporter	I	TGGTTTATCGTTGGTTTAA	1	D	A	S1	0.98	12	33	264
*slyB*	Outer membrane lipoprotein	I	TCGTTTAAGATTGGTTAAT	1	D	A	S1	0.88	12	32	100
*pmrD*	Connector of two-component systems	I	CTATTGCCGTTTTGTTTAT	1	D	A	S2	0.92	12	31	27
*orgB*	Effector protein of the SPI-1 type III secretion system	I	TTATTGAGGAGGCATTGAA	1	D	A	S3	0.80	12	32	20
*yobG*	Putative inner membrane protein	II	ACAGTTACTCCTGGTTTAA	2	D	A	S2	0.66	12	31	26
			GTTTTTAGGAATGATTCAC		R	A	S3	0.61		62	
*virK*	Antimicrobial peptide resistance	II	TCGTTGCCTTTACGTTTAA	2	D	A	S1	0.77	12	32	30
	protein		CCATTGATAAACTGTTTAA		D	R	S2	0.94		74	
*mig-14*	Putative transcriptional activator	II	ACATTTTTATTTGGTTAAG	2	D	A	S2	0.60	12	33	27
			ATGTTTAGCTTGTATTTAA		R	A	S3	0.98		57	
*ybjX*	Cationic peptide resistance protein	II	GTATTGACGATTGGTTAAT	2	D	A	S2	0.78	12	31	25
			TTGTTTAGATACGGTTTAC		R	A	S1	0.92		77	
*pcgL*	d-ala-d-ala dipeptidase	II	ATTTTAACCATCTGTTTAA	2	D	A	S2	0.79	12	30	47
			GAGTTTATATTTTGCTTAT		R	R	S3	0.58		90	
*ssrB*	Response regulator	II	ACATTAAAAGGCTGTTTAG	2	D	A	S2	0.80	12	32	150
			TGGTGTAGTTTTTGAAGAT		R	R	S2	0.55		−102	
*rstA*	Response regulator	III	TCGTTTAGAAAAGATTTAT	1	D	A	S1	0.85	23	42	21
*ompX*	Outer membrane protein	III	CGGTTGAGGGTTCGTTGAA	1	D	A	S1	0.86	21	42	237
*pagP*	Outer membrane lipid A acylase	IV	CTGTTTATAGTTTGTTAAG	2	D	A	S1	0.82	23	43	16
			TTTGTGAAAGCTTATTAAG		D	R	S3	0.60		126	
*pagD*	Putative outer membrane protein	IV	TGGTTAACTCTTCGTTGAA	2	D	A	S2	0.58	22	42	39
			GTGTTTAGAGAGAATTTAC		D	R	S3	0.89		144	
*iraP*	Regulator of RpoS stability	IV	CCGTTACGATATGGTTTAA	2	D	A	S1	0.60	21	39	218
			TTGTTTTTTGATGGTTTAT	2	D	R	S1	0.75		−53	
*ugtL*	Inner membrane protein that modifies the LPS	V	CGGTTGAGCAACTATTTAC	2	R	A	S3	0.73	30	51	176
			AATAATACTTTTAGTTTAA		R	R	S3	0.56		2	
*pagK*	Protein delivered to eukaryotic cells via outer membrane vesicles	V	CCATTTATAAAATATTTAA	2	R	A	S3	0.70	37	55	57
			ACGTTTAATATCTATAGTA		D	R	S3	0.60		−2	
*pgtE*	Outer membrane protease	V	ATTTTTACCTTATATTGAA	2	R	A	S3	0.60	49	68	45
			ATGATTATAGATTGCTTAT		D	R	S3	0.55		119	
*mgtC*	Inner membrane virulence protein that aids growth in low Mg^2+^	V	CTGTTTAAGTTTGTTTGAT	2	R	A	S1	0.72	48	67	284
			ATGTTTAAACACGCTTTAT		D	R	S1	0.72		−301	
			ATGTTTCCTTATATTTTAA		D	R	S3	0.60		−159	
*pagC*	Outer membrane protein	V	GTGTTTAGAGAGAATTTAC	2	R	A	S3	0.89	47	67	559
			TTATTTACGGTGTGTTTAA		R	R	S2	0.88		−527	
*pipD*	Pathogenicity island-encoded protein D	V	TTATTGAGGTTGTATTGAT	2	R	A	S3	0.83	58	76	40
			CCGTTACGCTGCTGGGTAT		D	R	S2	0.55		−55	

**Fig. 1:**
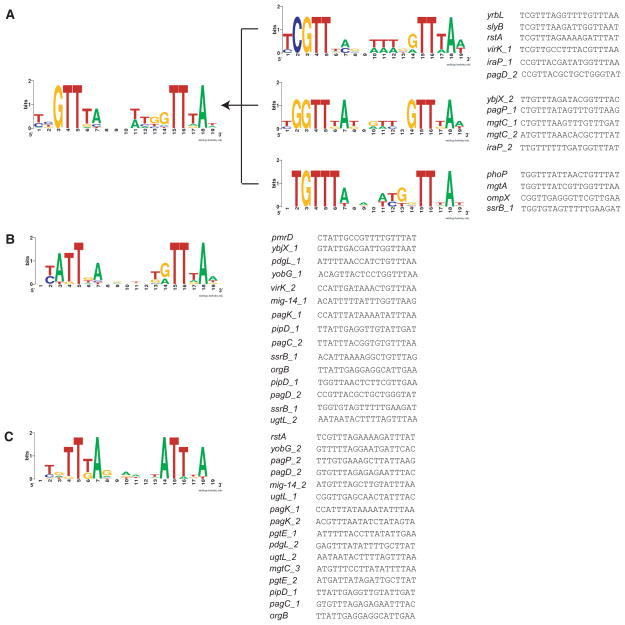
Subclasses of the PhoP box consensus sequence (submotifs). These subclasses were identified based on footprinting data (Table 1; Fig. S3) as described in Harari *et al*. (2010) and Zwir *et al*. (2005), where one sequence can belong to more than one cluster, and a subset of sequences can be hierarchically organized based on their specificity and sensitivity. A. Submotifs corresponding to the canonical direct repeat consensus sequence (S1). Three different subpatterns were identified within this class. B. Submotif S2 corresponding to a conserved variant in the first repeat sequence. C. Submotif S3 corresponding to a conserved variant in the second repeat sequence.

**Fig. 2:**
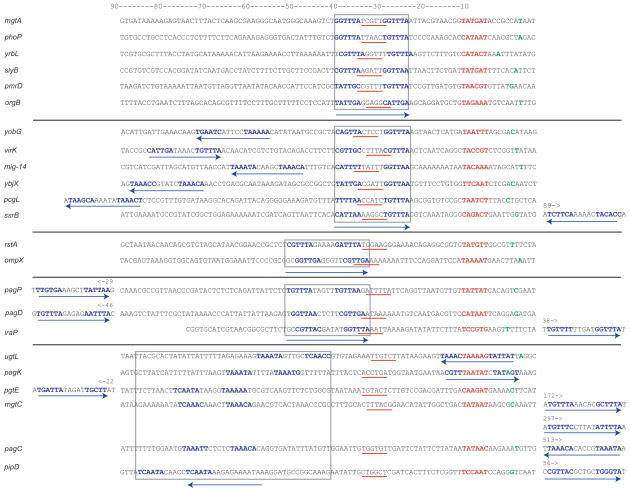
Architecture and DNA sequences of PhoP-activated promoter regions. DNA sequences corresponding to the 23 promoters analysed in this study. To facilitate the comparison of the 23 promoters, we aligned the corresponding sequences with respect to the predicted −10 hexamer using the 5′ most edge of this element as a referential landmark instead of the typically used transcription start site (Gaston *et al*., 1990; Liu *et al*., 2004). The transcription start site identified in Fig. S1 is indicated in green. (Transcription start sites for the *pipD* and *rstA* promoters are also included, data not shown.) This includes S1 nuclease protection data previously reported for *phoP* (Soncini *et al*., 1995; Garcia Vescovi *et al*., 1996), *pmrD* (Kato *et al*., 2003) and *orgB* (Aguirre *et al*., 2006) promoters as well as primer extension data reported for *mgtA* (Lejona *et al*., 2003), *iraP* (Tu *et al*., 2006) and *ssrB* (Feng *et al*., 2003; Bijlsma and Groisman, 2005) promoters. The PhoP box corresponding to regions protected by PhoP-P shown in Fig. S3 is indicated in blue. This also includes DNase I footprinting analysis previously reported for *phoP* (Lejona *et al*., 2003), *ugtL* (Shi *et al*., 2004), *pagC* (Perez *et al*., 2008), *pmrD* (Kato *et al*., 2003), *ssrB* (Bijlsma and Groisman, 2005), *iraP* (Tu *et al*., 2006) and *orgB* (Aguirre *et al*., 2006) promoters [as well as electrophoretic mobility shift assays (EMSA) analysis reported for the *mgtA* promoter (Lejona *et al*., 2003), and for the downstream PhoP box in the *pagC* promoter (this work, data not shown)]. The PhoP box orientation is indicated with blue arrows. The predicted −35 hexamer is underlined in red and the predicted −10 hexamer is indicated in red (Table 2). Activation PhoP boxes sharing common patterns including orientation, phasing and/or location with respect to the RNAP are indicated with grey boxes. The promoters were divided into five classes based on their shared *cis*-acting features. From top to bottom: Architecture I, Architecture II, Architecture III, Architecture IV and Architecture V.

‘A’ designates the promoter architecture in Fig. 2; ‘#’ correspond to the number of PhoP box(es); ‘O’ corresponds to the PhoP box orientation where D = direct and R = reverse; submotif corresponds to the classes of PhoP box motifs in Fig. 1; Score/submotif correspond to the score within a submotif.

The PhoP protein binds to its DNA targets *in vivo* only when phosphorylated (Shin and Groisman, 2005; Shin *et al*., 2006). This process is controlled by the PhoQ protein (Groisman and Mouslim, 2006), which activates the PhoP protein when the bacterium experiences low extracytoplasmic Mg^2+^ (Garcia Vescovi *et al*., 1996), antimicrobial peptides (Bader *et al*., 2005) or acid pH (Prost *et al*., 2007). Phosphorylated PhoP (PhoP-P) binds to a hexanucleotide direct repeat separated by five nucleotides, designated the PhoP box, in its target promoters (Kato *et al*., 1999). This suggests that a PhoP-P dimer (Bachhawat and Stock, 2007) binds a PhoP box in a head to tail configuration. Among the relatively small number of PhoP-activated promoters experimentally defined to date, the PhoP box can be found at various distances and in both possible orientations with respect to the −10 hexamer sequence recognized by RNAP (Shi *et al*., 2004; Shin and Groisman, 2005; Zwir *et al*., 2005; Perez *et al*., 2008; Harari *et al*., 2010), indicative of PhoP utilizing different mechanisms to promote gene transcription (Barnard *et al*., 2004; Browning and Busby, 2004). Indeed, the C-terminal domain of the α subunit of RNAP is required for transcription of a subset of PhoP-activated promoters (Perez and Groisman, 2009). And the PhoP-activated DNA-binding protein SlyA is necessary to overcome the silencing effects of the histone-like nucleoid-structuring protein (H-NS) at other PhoP-dependent promoters (Kong *et al*., 2008; Perez *et al*., 2008; Song *et al*., 2008; Zhao *et al*., 2008).

Here we report the spectrum and organization of the *cis*-acting features present in PhoP-activated promoters (i.e. the promoter architectures), and the different roles that the PhoP protein plays at PhoP-activated promoters. Our analysis reveals that PhoP utilizes five distinct promoter architectures to drive transcription of its activated genes in *Salmonella*. These architectures are composed of specific (as opposed to arbitrary) combinations of *cis*-acting regulatory elements. Finally, we establish that *Salmonella* employs different promoter architectures to control ancestral and horizontally acquired PhoP-activated genes.

## Results

### Mapping the transcription start sites for PhoP-activated genes

We previously identified a large number of genes directly activated by the PhoP protein in *Salmonella* using expression microarray analysis of wild-type versus *phoP* mutant strains and chromatin immunoprecipitation (ChIP) experiments (Harari *et al*., 2010). However, except for a limited number of cases (Kato *et al*., 2003; Lejona *et al*., 2003; Shi *et al*., 2004; Bijlsma and Groisman, 2005; Zwir *et al*., 2005; Aguirre *et al*., 2006; Tu *et al*., 2006; Perez *et al*., 2008), the particular sequences recognized by the PhoP protein at the corresponding promoters, as well as the location of these sequences relative to the transcription start sites, have remained unknown. Therefore, to define the scope of PhoP-activated promoters, we first determined the transcription start sites for 15 genes known to be directly activated by the PhoP protein (Harari *et al*., 2010) by carrying out S1 mapping experiments with RNA harvested from isogenic wild-type and *phoP Salmonella* strains grown under PhoP-inducing (i.e. 10 µM Mg^2+^) and -repressing (i.e. 10 mM Mg^2+^) conditions. Then, we analysed their transcription start sites together with those corresponding to other previously mapped PhoP-dependent promoters.

A single start site was detected for most PhoP-activated genes including *yrbL*, *ompX, yobG, pcgL, pagP, pagD, virK, mig-14, pagK, pgtE, mgtC* (Fig. S1A–K), *pmrD* (Kato *et al*., 2003) and *orgB* (Aguirre *et al*., 2006). The corresponding promoters were active in the wild-type strain following growth in low Mg^2+^ but not in the *phoP* mutant regardless of the growth condition. A few PhoP-activated genes, however, have additional start sites that are PhoP-independent because they were observed in the *phoP* mutant strain whether grown in high or low Mg^2+^ as well as in the wild-type strain grown under non-inducing (i.e. high Mg^2+^) conditions (Fig. S1L and M). This is the case of the *ybjX* (Fig. S1L) and *iraP* (Tu *et al*., 2006) genes, as well as the *slyB* and *phoP* genes, which exhibit two transcription start sites both in *Salmonella* (Garcia Vescovi *et al*., 1996) (Fig. S1M) and in *Escherichia coli* (Minagawa *et al*., 2003). The *slyB* and *phoP* genes differ from the *ybjX* (Fig. S1L) and *iraP* (Tu *et al*., 2006) genes in that the S1 product that is PhoP-independent is present at higher levels after growth in high Mg^2+^ than in low Mg^2+^ in both wild-type and *phoP* mutant strains (Garcia Vescovi *et al*., 1996) (Fig. S1M). A single PhoP-dependent transcription start site was originally reported for the *mgtA* gene (Lejona *et al*., 2003), but it was later found that overexpression of the *rob* gene promoted *mgtA* transcription from a different site even in a strain deleted for the *phoP* and *phoQ* genes (Barchiesi *et al*., 2008).

### A subset of PhoP-activated mRNAs has unusually long 5′ leader regions

In bacteria, the median length of the 5′ leader region is 30 nt (Lynch *et al*., 2005; Pinto *et al*., 2011). Interestingly, it is > 50 bp for nine of the 23 analysed PhoP-dependent transcripts (i.e. *mgtA*, *slyB*, *ssrB*, *ompX*, *iraP*, *pagK*, *ugtL*, *mgtC* and *pagC*) (Table 1; Fig. S2A). This raises the possibility of these genes being subjected to additional regulatory inputs via their 5′ leader regions, which could affect mRNA stability, transcription elongation into the region and/or translation of the transcript. Indeed, the *mgtA* mRNA includes a 5′ leader that controls elongation into the coding region in response to the cytoplasmic Mg^2+^ (Cromie *et al*., 2006) and proline (Park *et al*., 2011) levels. Likewise, the 5′ leader region of the *mgtCBR* operon contributes to the Mg^2+^-dependent regulation of the corresponding coding regions (Spinelli *et al*., 2008). Finally, the levels of the PagC protein differ significantly between wild-type and *hfq* mutant *Salmonella* (Sittka *et al*., 2008), and this could be due to an sRNA acting on the 559-nt-long leader region of the *pagC* transcript.

### Correlation between PhoP box submotif and PhoP box orientation

We carried out DNase footprinting analysis of the promoter regions corresponding to 15 PhoP-activated genes utilizing phosphorylated PhoP-His6 protein (i.e. PhoP-P). The PhoP-P protein protected sequences of different lengths within each promoter (e.g. 20 nt in the *rstA* promoter and > 30 nt in the *virK* and *mig-14* promoters), most of which cover both repeats that comprise the PhoP box [i.e. sequences resembling the hexanucleotide direct repeat (T/G)GTTTA separated by five nucleotides (Kato *et al*., 1999)]. A single region was protected in four promoters (Fig. S3A–D), whereas two sites were protected in the remaining 11 promoters (Fig. S3E–O). In the latter case, PhoP-P exhibited differential affinity for the two sites (Fig. S3E–O). The occurrence of two PhoP boxes had been previously established for the *ugtL* (Shi *et al*., 2004) and *iraP* (Tu *et al*., 2006) promoters. Here, we generalize our previous findings and bioinformatics analysis (Harari *et al*., 2010) to 23 promoters including those driving transcription of the *pagC*, *mgtC*, *pagK*, *pagP*, *pgtE*, *pipD*, *virK*, *ybjX*, *pcgL, yobG* and *pagD* genes where the second PhoP box had not been detected in previous experimental studies (Lejona *et al*., 2003; Zhao *et al*., 2008).

The sequences corresponding to the PhoP boxes reflect, on average, the consensus motif (T/G)GTTTA 5 nt (T/G)GTTTA. Based on our DNase footprinting analysis, we redefined distinct subclasses of this consensus – termed PhoP box submotifs – originally established in PhoP-activated promoters (Zwir *et al*., 2005; Harari *et al*., 2010) (Fig. 1). For example, the single PhoP box sequence in the *phoP*, *mgtA*, *yrbL*, *ompX* and *slyB* promoters resembles the canonical direct repeat (Table 1), which can be further decomposed into three subclasses when considering sequences from other promoters (i.e. (C/T/G)GTTTA, and/or conserved peripheral nucleotides) (Fig. 1A). In the case of the *ybjX*, *pcgL*, *yobG*, *virK*, *mig-14* and *ssrB* promoters (Table 1), one of the two PhoP boxes is located in the direct orientation (i.e. same orientation as the single PhoP box in the *mgtA* promoter) and displays a different consensus sequence in the first repeat (i.e. (T/C)ATTTA) (Fig. 1B). Conversely, one of the PhoP boxes in the *yobG*, *mig-14*, *ugtL*, *pagK*, *pgtE*, *pagC* and *pipD* promoters is located in the opposite relative orientation as the single PhoP box in the *mgtA* promoter (Table 1) and exhibits a slightly different consensus sequence in the second repeat (i.e. TATTTA) (Fig. 1C). Overall, the PhoP box submotifs correlate well (*F* statistics, *P*-value < 0.005, see *Experimental procedures*) with the orientation of the PhoP box.

### The architectural diversity of PhoP-activated promoters

We analysed 23 PhoP-activated promoters whose transcription start sites (Fig. S1) (Soncini *et al*., 1995; Garcia Vescovi *et al*., 1996; Feng *et al*., 2003; Kato *et al*., 2003; Lejona *et al*., 2003; Bijlsma and Groisman, 2005; Aguirre *et al*., 2006; Tu *et al*., 2006) and PhoP boxes (Fig. S3) (Kato *et al*., 2003; Lejona *et al*., 2003; Shi *et al*., 2004; Bijlsma and Groisman, 2005; Aguirre *et al*., 2006; Tu *et al*., 2006; Perez *et al*., 2008) had been experimentally determined (Table 1; Fig. 2). This analysis entailed inspecting the promoter sequences for the following features: (i) the number of PhoP boxes, (ii) the location of the PhoP box(es) defined as the distance between the PhoP box and the −10 hexamer sequence predicted to be recognized by RNAP, (iii) the orientation of each PhoP box relative to the predicted −10 hexamer sequence, where direct orientation refers to the half PhoP box sequence 5′ (T/G)GTTTA 3′ pointing towards the −10 hexamer sequence, and reverse orientation where the half PhoP box sequence is pointing away from the predicted −10 hexamer sequence, (iv) the PhoP box rotational phasing along the DNA with respect to the predicted −10 hexamer, and (v) the predicted sequence elements characteristic of promoters recognized by the σ^70^ form of RNAP, including the −10 hexamer, the −35 hexamer and the spacer length between the two hexamers (Barnard *et al*., 2004; Shultzaberger *et al*., 2007) (Fig. 2). Our analysis revealed the presence of five distinct architectures in PhoP-activated promoters (Table 1; Fig. 2).

Architecture I, present in the *mgtA*, *phoP*, *yrbL*, *slyB*, *pmrD* and *orgB* promoters, harbours a single PhoP box in the direct orientation located 12 nt upstream of the predicted −10 hexamer (except for the *yrbL* promoter where the distance is only 11 nt), i.e. one turn of the DNA helix away from this hexamer. Architecture II, present in the *yobG*, *pcgL*, *virK*, *mig-14*, *ybjX* and *ssrB* promoters, contains two PhoP boxes, one in the direct orientation with location and phasing similar to the single PhoP box in promoters with architecture I, and another PhoP box located upstream of this site (except for the *ssrB* promoter where the second PhoP box is located downstream of the first PhoP box). Architecture III, present in the *rstA* and *ompX* promoters, includes a single PhoP box in the direct orientation located 21–23 nt upstream of the −10 hexamer, i.e. two turns of the DNA helix away from this hexamer. Architecture IV, present in the *pagP*, *pagD* and *iraP* promoters, carries two PhoP boxes, one in the direct orientation with location and phasing similar to the single PhoP box in architecture III promoters, and another PhoP box located either upstream or downstream of this site. Architecture V, present in the *ugtL*, *pagK*, *pgtE*, *mgtC*, *pagC* and *pipD* promoters, also displays two PhoP boxes, one in the reverse orientation, located 30, 37, 49, 48, 47 or 58 nt upstream of the −10 hexamer, separated by half or near half-integral turns of the DNA helix from this hexamer. The other PhoP box either overlaps the −10 hexamer or is located downstream of the transcription start site.

There is a cross-correlation between PhoP box submotif and promoter architecture (*F* statistics, *P*-value < 0.01). For instance, promoters with architectures I, III and IV harbour primarily PhoP boxes with sequences resembling submotif S1 (Fig. 1A). By contrast, promoters with architectures II and V mainly harbour PhoP boxes resembling submotifs S2 (Fig. 1B) and S3 (Fig. 1C) respectively.

### PhoP uses different mechanisms to activate transcription from promoters harbouring a single PhoP box

Activator proteins promote gene transcription in at least two ways depending on the location of their binding sites relative to those of the RNAP (Barnard *et al*., 2004; Browning and Busby, 2004). In Class I activation, the activator binds to a sequence located upstream of the −35 hexamer and recruits RNAP by interacting with the C-terminal domain of its α subunit (α-CTD). In Class II activation, the activator binds to a sequence that overlaps with the −35 hexamer, and the interaction is established with the σ subunit of RNAP, although additional RNAP subunits may also be contacted.

**Fig. 3:**
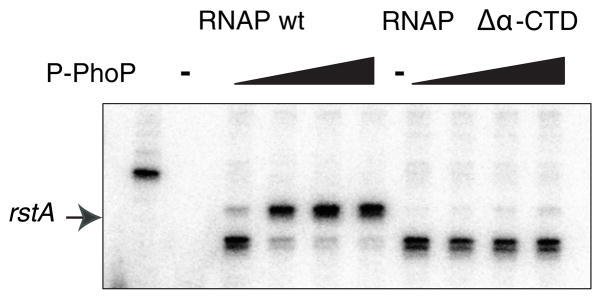
The α-CTD subunit of RNA polymerase is required to promote transcription from the *rstA* promoter. Single-round *in vitro* transcription assays with linear templates corresponding to the *rstA* promoter using wild-type RNAP or mutant alpha Δ235 (αΔ-CTD) RNAP, and increasing amounts of phosphorylated *Salmonella* PhoP-His-6 protein (0, 65, 130 and 325 nM). Arrows show PhoP-dependent transcripts. Additional bands correspond to spurious transcripts that are not PhoP-activated.

The single PhoP box in the *mgtA*, *phoP*, *yrbL*, *slyB*, *pmrD* and *orgB* promoters is located in the direct orientation *c*. 12 nt upstream of the predicted −10 hexamer (architecture I; Fig. 2). These promoters are predicted to behave as Class II promoters because their respective PhoP boxes overlap the predicted −35 hexamer (Table 2; Fig. 2). This was demonstrated for the *mgtA* promoter because the α-CTD of RNAP is not required for PhoP-dependent activation of *mgtA in vitro* (Perez and Groisman, 2009). The *ompX* and *rstA* promoters (architecture III; Fig. 2) also harbour a single PhoP box in the direct orientation. However, the PhoP box is located 21 nt and 23 nt upstream from the predicted −10 hexamer in the *ompX* and *rstA* promoters respectively. This is one turn of the DNA helix upstream from the location of the PhoP box in the architecture I promoters (Fig. 2). We determined that the α-CTD subunit of RNAP is required to promote PhoP-dependent activation of transcription from the *rstA* gene (Fig. 3). The production of spurious transcripts by the alpha Δ-235 (αΔ-CTD) RNAP indicates that this mutant RNAP is competent for transcription. The bands below the *rstA* transcripts, likely originating from cryptic sites, are commonly observed in *in vitro* transcription assays, particularly when naked linear DNA is used as template. The spurious transcripts originate within 20 nt of the *rstA* transcription start site. The data indicate that PhoP and RNAP most likely occlude these cryptic sites to favour *rstA* transcription. PhoP and mutant RNAP, on the other hand, are unable to promote *rstA* transcription and, thus, do not occlude the cryptic sites. The spurious transcripts observed in the two lanes where the PhoP protein was omitted demonstrate that both wild-type and mutant RNAP are able to promote gene transcription (i.e. the inability of the mutant RNAP to promote PhoP-dependent *rstA* transcription cannot be due simply to the enzyme being dead). In addition, we have demonstrated that same preparation of mutant RNAP could promote transcription from the *mgtA* promoter (Perez and Groisman, 2009). Cumulatively, these data indicate that the location of the single PhoP box affects the mechanism by which PhoP promotes gene transcription.

**Table 2:** Summary of the RNAP features in 23 PhoP-activated promoters

	Distance	Sequence	Mismatches	Sequence	Mismatches	Spacer length	RNAP
Promoter	−10 to +1	−10	−10	−35	−35	−35 to −10	Score
*mgtA*	7	TATGAT	1	TCGTTG	4	17	0.74
*phoP*	7	CATAAT	1	TTAACT	2	17	0.82
*yrbL*	2	CATACT	2	TAGGTT	4	18	0.62
*slyB*	6	TATGAT	1	AAGATT/TTAAGA	4/2	18/20	0.68/0.68
*pmrD*	5	TAACGT	3	CCGTTT/TTGCCG	5/2	18/21	0.53/0.53
*orgB*	7	TAGAAA	2	GAGGCA	3	16	0.68
*yobG*	5	TAATTT	3	TACTCC/GTTACT	4/3	19/21	0.56/0.54
*virK*	6	TACCGT	3	TTTACG	2	16	0.70
*mig-14*	7	TACAAA	2	TTTATT	3	19	0.65
*ybjX*	5	TTCAAT	2	ACGATT/TTGACG	4/1	18/21	0.60/0.60
*pcgL*	4	TAATCT	3	ACCATC/TAACCA	5/4	18/20	0.56/54
*ssrB*	6	CAGACT	3	AAGGCT	4	17	0.61
*rstA*	5	TATGTT	2	TGGAAG	3	17	0.75
*ompX*	7	TAAAAT	1	TTGAAA	1	19	0.79
*pagP*	6	TATTAT	1	ATTTTA/AAGATT	4/4	16/19	0.69/0.68
*pagD*	6	CATAAT	1	AATAAA/TGAATA/TTGAAT	4/3/2	17/19/20	0.72/0.70/0.66
*iraP*	4	TCCGTG	5	TTAAAT/TTTAAA	3/2	18/19	0.53/0.54
*ugtL*	7	TAAAAG	2	TTGTCT/TAGAAA	3/3	15/21	0.66/0.64
*pagK*	4	TAATAT	2	CTGATG/ACCTGA	3/5	15/17	0.65/0.61
*pgtE*	5	CAAGAT	3	TGTACT	3	17	0.68
*mgtC*	5	TATAAT	0	TTTACG	2	16	0.83
*pagC*	6	TATAAC	1	TGGTGT	4	17	0.71
*pipD*	5	TCCAAT	2	CTGGCT	3	17	0.72

The RNAP score of a query sequence is based on its similarity with the RNAP consensus pattern (−10 sequence, −35 sequence and the distance between them) identified from examples in the RegulonDB database (Harari *et al.*, 2009).

### PhoP-activated promoters with two PhoP boxes

In contrast to the eight promoters discussed above, the remaining 15 promoters (i.e. corresponding to the *yobG, pcgL, virK, mig-14, ybjX, ssrB, pagP, pagD, iraP, ugtL, pagK, pgtE, mgtC, pagC* and *pipD* genes) harbour two PhoP boxes, which can be found in the direct or reverse orientations (Table 1; Fig. 2). One of the PhoP boxes is invariably located at or farther upstream of the site normally occupied by the predicted −35 hexamer (Fig. 2). In nine of these promoters (i.e. *yobG, pcgL, virK, mig-14, ybjX, ssrB, pagP, pagD* and *iraP*), corresponding to architectures II and IV, one of the PhoP boxes is in the same orientation, location and phasing as that present in promoters with architectures I and III respectively. Promoters with architectures II and IV differ from promoters with architectures I and III in harbouring a second PhoP box (Fig. 2). In architecture V promoters (i.e. *ugtL, pagK, pgtE, mgtC, pagC* and *pipD*), one of the PhoP boxes is located farther upstream from the location of the single PhoP box in the promoters with architectures I and III (Table 1; Fig. 2). This PhoP box is invariably in the reverse orientation and separated by half-integral turns from the predicted −10 hexamer (Table 1; Fig. 2), which distinguishes these promoters from architecture II and IV promoters.

The location of the second PhoP box varies among promoters with architectures II, IV and V (Fig. 2). For all architecture V promoters except the *pgtE* promoter, the location of the second PhoP box suggests it functions as a repression site despite being part of PhoP-activated promoters. For example, the second PhoP box in the *ugtL* promoter overlaps with the predicted −10 hexamer (Fig. 2), suggesting that PhoP-P binding to this sequence likely blocks transcription initiation. In the case of the *mgtC* promoter, the second PhoP box is downstream of the mapped transcription start site (Fig. 2), implying it operates as a roadblock to hinder transcription elongation (Browning and Busby, 2004).

The cross-correlation between the sequence of the PhoP box and its predicted role reveals that PhoP boxes predicted to participate in activation display primarily the S1 and S2 submotifs whereas those anticipated to act in repression display mainly the S3 submotif (Table 1; Fig. 1; *F* statistics, *P*-value < 0.008).

### The location and orientation of a PhoP box are critical for PhoP-promoted transcription

**Fig. 4:**
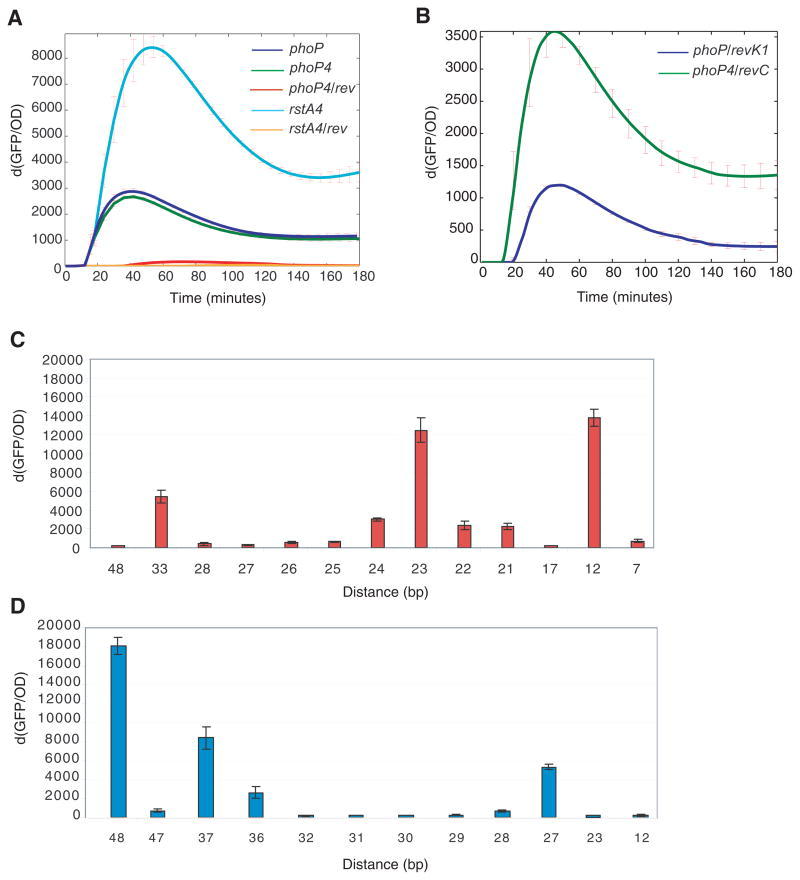
Transcription of PhoP-activated promoters is affected by the location, orientation and phasing of the PhoP box. GFP expression produced by *Salmonella* harbouring plasmids with a promoterless *gfp* gene driven by derivatives of the *phoP* promoter. Each construct is a shortened derivative of the corresponding PhoP-activated promoters that includes the most upstream PhoP box and the RNAP binding site. The resulting DNA fragment was cloned in front of promoterless *gfp* gene in a plasmid pMS201 and introduced into a *Salmonella* strain expressing an HA-tagged PhoP (A and B) from its normal chromosomal location (EG13918) or wild-type PhoP (C and D). Cells were grown in N-minimal media containing 10 mM MgCl_2_ and then switched to media containing 50 µM MgCl_2_. Promoter activity (*y*-axis) was measured by normalizing GFP expression to cell density and pre-proceeded as described in *Experimental procedures*. Shown are the mean and S.D. values of at least three independent experiments. A. The *phoP* (blue line) promoter compared with its shortened derivative *phoP4* (green line). The *phoP4* (green line) and *rstA4* (cyan line) promoters compared with their derivatives *phoP4/rev* (red line) and *rstA4/rev* (orange line), respectively, where the PhoP box orientation was reversed from their original locations. B. The *phoP4/revK* (blue line) and the *phoP4/revC* (green line) promoters, where the orientation of the PhoP box was reversed and moved to positions of the PhoP box in the natural *pagK* (blue line) and *mgtC* (green line) promoters (see Fig. 2). C. The PhoP box moved upstream/downstream from its normal location and orientation in the *phoP4* promoter at 7, 12, 17, 21, 22, 23, 24, 25, 26, 27, 28, 33, 36 and 48 bp from the predicted −10 hexamer. D. The oppositely oriented PhoP box in the *phoP4* promoter was moved upstream/downstream at 12, 23, 27, 28, 29, 30, 31, 32, 36, 37, 47 and 48 bp from the predicted −10 hexamer.

To define the combinations of PhoP box location and orientation that enable PhoP-dependent gene transcription, we measured the fluorescence produced by strains harbouring engineered plasmids carrying fusions between wild-type or mutant PhoP-activated promoters and a promoterless *gfp* gene. Promoters labelled with the suffix ‘4’ (e.g. *phoP4*) are shortened derivatives of the natural promoters that recapitulate PhoP-dependent gene transcription. They include the minimum DNA sequence corresponding to the PhoP box(es) identified in DNase footprinting assays (Fig. S3) and to the −10 and −35 hexamers predicted from the S1 mapping experiments (Fig. S1). For example, cells harbouring a plasmid with the promoter *phoP4*, containing only 52 nt upstream and 5 nt downstream of the PhoP-dependent transcription start site, displayed a behaviour that was virtually identical to that of cells harbouring a plasmid with a longer PhoP promoter fragment that included 112 additional bp upstream (Fig. 4A). Therefore, all the sequence information necessary for Mg^2+^-regulated PhoP-dependent transcription appears to be present in the cloned 58 bp DNA fragment.

We believe that PhoP is initiating transcription from the *phoP4* promoter from the same location as when the promoter is in the chromosome (as opposed to utilizing alternative −10 sequences). This is because mutation of the predicted −10 hexamer significantly compromised expression (Fig. S4).

Classical promoter enhancers retain the ability to activate gene transcription when placed in the opposite orientation (Kadener *et al*., 2002). Thus, we tested whether a PhoP box that is normally in one orientation can function when present at the same position but in the opposite orientation. We made derivatives of the natural *phoP* and *rstA* promoters where the 17 nucleotides corresponding to each of their single PhoP boxes in the direct orientation were placed in the opposite orientation (*phoP4-rev* and *rstA4-rev*). The wild-type strain harbouring the resulting plasmids produced no fluorescence (Fig. 4A), in contrast to the fluorescence displayed by the strains harbouring the isogenic plasmids with the original *phoP* and *rstA* promoters (Fig. 4A). These data demonstrate that the PhoP box functions in an orientation-dependent manner, at least when located 12 nt or 23 nt upstream of the −10 hexamer sequence of RNAP. Importantly, the PhoP box present in the *phoP* promoter is competent to mediate transcription when present in the reverse orientation at distances from the predicted −10 region found in natural PhoP-activated promoters. For example, the single PhoP box from the *phoP* promoter could functionally replace the PhoP box in the *pagK* promoter located 37 nucleotides from the predicted −10 region (*phoP-revK1*, Fig. 4B), and the PhoP box in the *mgtC* promoter located 48 nucleotides from the predicted −10 region (*phop-revC*, Fig. 4B).

We then explored the range of distances over which a directly oriented PhoP box can promote gene activation. The PhoP box mediated transcription when located 12, 23 or 33 nt upstream of the predicted −10 hexamer (Fig. 4C). Similar fluorescence values were produced by strains carrying constructs with the *phoP* PhoP box 12 or 23 nt upstream of the predicted −10 hexamer (Fig. 4C), which correspond to the normal locations of the PhoP box in the *phoP* and *rstA* promoters respectively (Fig. 2). The PhoP box could still drive *gfp* transcription when located 33 nt upstream of the −10 hexamer even though there are no examples of natural PhoP-activated promoters with a directly oriented PhoP box at this location (Fig. 4C). Yet, the strain with the latter construct produced only half as much fluorescence as the strains with the former two plasmids (Fig. 4C).

The three promoters described above share the orientation and sequence of the PhoP box. However, they differ in the location of the PhoP box, which, curiously, is one or two integral turns of a DNA helix away from the position of the PhoP box in the other promoters. This suggests that the PhoP-P protein promoting transcription from these three promoters is positioned on the same face of the DNA. If being on the same face of the DNA is critical for PhoP-activated gene transcription, then altering the distance by a number of nucleotides different from 10–11 (corresponding to one turn of a DNA helix) should abolish gene transcription. As predicted, strains harbouring plasmids where *gfp* transcription was driven by a promoter with a directly oriented PhoP box located 7, 17 or 28 nt upstream of the predicted −10 hexamer sequence produced little fluorescence (Fig. 4C). Similarly low levels of fluorescence were displayed by strains where the PhoP box was situated 25, 26 or 27 nt upstream from the predicted −10 hexamer sequence (Fig. 4C). And when the PhoP box was positioned 21, 22 or 24 nt upstream from the predicted −10 hexamer, expression was ∼ 25% of that observed when the PhoP box was at position 23 (Fig. 4C).

Next, we examined the locations at which the PhoP box could promote gene transcription when present in the reverse orientation. We created a set of promoters analogous to those described above, except that the PhoP box was present in the reverse orientation at various distances from the −10 hexamer, including some corresponding to the location of natural PhoP-dependent promoters (Fig. 2). The PhoP box promoted transcription when placed 27, 37 or 48 nt upstream of the predicted −10 hexamer (Fig. 4D). Interestingly, expression increased as the distance of the PhoP box to the −10 hexamer increased (Fig. 4D). Consistent with the notion that being on a particular face of the DNA is necessary for PhoP-promoted gene transcription, there was little expression when the PhoP box was present 23, 28, 29, 30, 31 or 32 nt upstream of the −10 hexamer sequence (Fig. 4D). In contrast to promoters harbouring the PhoP box in the direct orientation, the PhoP box in the three promoters with the PhoP box in the opposite orientation is separated by half-integral turns of the DNA helix from the predicted −10 hexamer. These results support the idea that it is the combination of location and orientation of the PhoP box that determines on which face of the DNA helix PhoP-P is found and the ability of PhoP-P bound to such PhoP box to establish productive interactions with RNAP.

### A second PhoP box modulates transcription in a promoter with two PhoP boxes

**Fig. 5:**
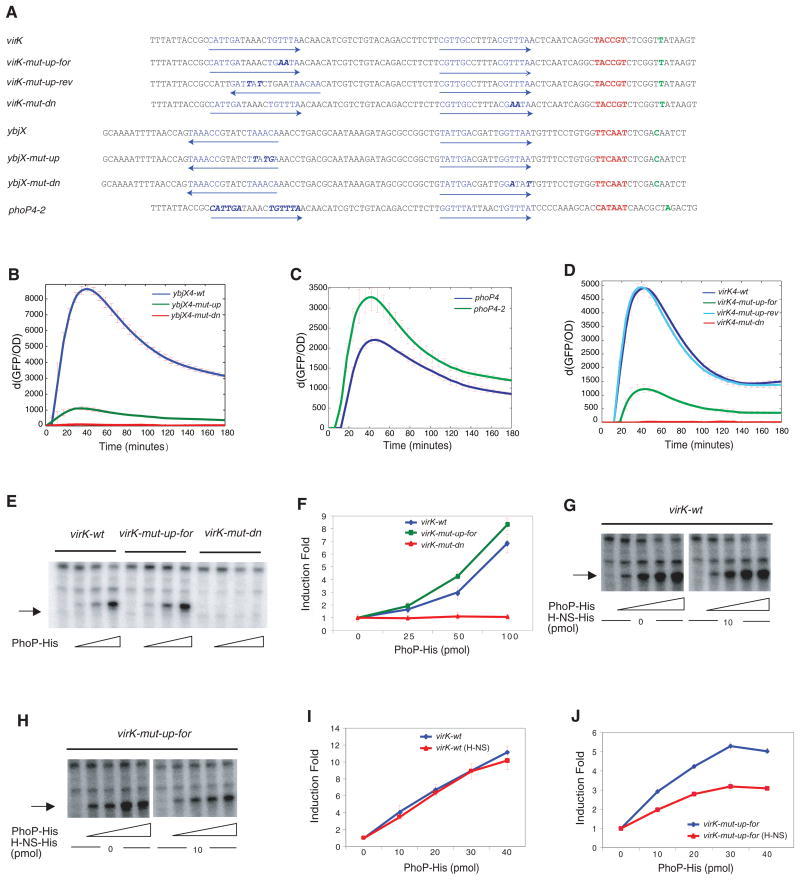
Different roles for the two PhoP boxes in PhoP-activated promoters with two PhoP boxes. A. DNA sequences of a set of natural and mutant PhoP-activated promoters. All constructs harbour the predicted −10 and −35 hexamers identified in the S1 mapping experiments (Fig. S1). The predicted −10 sequences and the transcription start sites are indicated in red and green respectively (Table 2). PhoP boxes and their orientation are indicated in bold and blue respectively (Table 1). Nucleotide substitutions introduced in the PhoP boxes are indicated in bold italics. Cells in all GFP expression experiments were grown in N-minimal media containing 10 mM MgCl_2_ and switched to media with 50 µM MgCl_2_. The promoter activity (*y*-axis) was determined by normalizing GFP expression to cell density and pre-processed as described in *Experimental procedures*. Shown are the mean and S.D. values of at least three independent experiments. B. GFP expression displayed by a *Salmonella* strain expressing HA-tagged PhoP from its normal chromosomal location (EG13918) harbouring plasmids with a promoterless *gfp* gene driven by the wild-type *ybjX* promoter (blue line) or derivatives *ybjX-mut-dn* (red line) and *ybjX-mut-up-rev* (green line) where the two PhoP boxes were individually mutated. C. GFP expression displayed by a *Salmonella* strain expressing HA-tagged PhoP from its normal chromosomal location (EG13918) harbouring plasmids with a promoterless *gfp* gene driven by the *phoP4* promoter (blue line) or its derivative *phoP4-2* (green line), with a second PhoP box 26 nt upstream of the first PhoP box. D. GFP expression displayed by a *Salmonella* strain expressing HA-tagged PhoP from its normal chromosomal location (EG13918) harbouring plasmids with a promoterless *gfp* gene driven by the wild-type *virK* promoter (blue line) or derivatives *virK-mut-dn* (red line), virK*-mut-up-for* (green line) and *virK-mut-up-rev* (cyan line) where the two PhoP boxes were individually mutated. E. Run-off *in vitro* transcription assays with linear templates corresponding to the *virK* promoter region (*virK-wt*), and an equivalent DNA fragment with point mutations in the first (*virK-mut-dn*) and second (*virK-mut-up-for*) PhoP boxes as indicated in (A), RNAP and increasing amounts of PhoP-His protein (0, 25, 50 and 100 pmol). The transcripts are indicated with arrows. Shown are the mean and S.D. values of at least two independent experiments. F. Quantification of the *in vitro* transcription assays in (E), where the induction fold was calculated with respect to samples lacking PhoP-His protein. G and H. Run-off *in vitro* transcription assays with linear templates corresponding to the *virK* promoter region (*virK-wt*) and an equivalent DNA fragment with point mutations in the second PhoP box (*virK-mut-up-for*) as indicated in (A), RNAP, PhoP-His protein only (0, 10, 20, 30 and 40 pmol) and in combination with H-NS (0 and 10 pmol). The transcripts are indicated with arrows. Shown are the mean and S.D. values of at least two independent experiments. I and J. Quantification of the *in vitro* transcription assays in (G) and (H), where the induction fold was calculated with respect to samples lacking PhoP-His protein. Blue and red lines indicate transcripts with PhoP protein only and in combination with H-NS respectively.

Most promoters with architectures II and IV harbour two PhoP boxes located at the position corresponding to the predicted −35 hexamer and/or upstream of it, suggesting the PhoP binding sites operate as activation sites (Fig. 2). To test this hypothesis, we measured the fluorescence produced by wild-type *Salmonella* harbouring a medium copy number plasmid with a promoterless *gfp* gene driven by the natural architecture II *ybjX* promoter or by derivatives with nucleotide substitutions in each of the two PhoP boxes (Fig. 5A). Nucleotide substitutions in the PhoP box proximal to the predicted −10 hexamer abolished expression (*ybjX4-mut-dn*; Fig. 5B). By contrast, mutation of the upstream PhoP box, which is in the reverse orientation and located 30 nt upstream of the first PhoP box (Fig. 5A), dramatically reduced fluorescence (i.e. > 8-fold) but did not eliminate it (*ybjX4-mut-up-rev*; Fig. 5B). Because having a second PhoP box upstream of a PhoP box located 12 nt upstream of the −10 hexamer increases gene transcription in the *ybjX* promoter, we wondered whether adding a second PhoP box to a promoter that normally has a single PhoP box increases its expression levels. As hypothesized, adding a second PhoP box 26 nt upstream of the single PhoP box in the *phoP* promoter, which is the location of the architecture II *virK* promoter (Fig. 2), enhanced its transcription levels originating from the engineered promoter (*phoP4-2*; Fig. 5C).

Architecture V promoters also harbour two PhoP boxes, one of which is located at the position corresponding to the −10 hexamer or downstream of the transcription start site, suggesting they operate as repression sites (Fig. 2). This is the case in the *ugtL* promoter, which harbours one PhoP box overlapping the −10 sequence (Shi *et al*., 2004) and is anticipated to function as a repression site despite being part of a PhoP-activated promoter.

### A PhoP box necessary to overcome silencing by H-NS

Why are two PhoP boxes needed for activation in architecture II and IV promoters instead of a single high-affinity site for PhoP-P? A possible explanation is that one of the PhoP boxes has a role(s) other than interacting with RNAP. For example, transcription of many horizontally acquired genes, including some that are PhoP-activated, is silenced by nucleoid-associated proteins (Dorman, 2007; Perez *et al*., 2008). This raises the possibility of the second PhoP box being involved in overcoming gene silencing.

We chose as a model for this group of promoters the architecture II *virK* promoter, which harbours two PhoP boxes (Fig. 2) and is silenced by the H-NS protein (Navarre *et al*., 2006). Then, we measured the fluorescence produced by wild-type *Salmonella* harbouring a medium copy number plasmid with a promoterless *gfp* gene driven by the natural *virK* promoter or by derivatives with nucleotide substitutions in one or the other PhoP box (Fig. 5A). Nucleotide substitutions in the PhoP box proximal to the predicted −10 hexamer abolished expression (*virK4-mut-dn*; Fig. 5D). By contrast, mutation of the second PhoP box in the *virK* promoter, which is in the direct orientation and located 26 nt upstream of the first PhoP box (Fig. 5A), dramatically reduced fluorescence but did not eliminate it (*virK4-mut-up-for*; Fig. 5D). These behaviours mimic those exhibited by derivatives of the *ybjX* promoter with mutations in each of its two PhoP boxes (Fig. 5B).

We further evaluated the *virK* promoter by carrying out *in vitro* transcription reactions with wild-type or mutant DNA templates, purified PhoP protein and RNAP. In agreement with the *in vivo* results, there was no transcription when the *virK-mut-dn* mutant DNA was used (Fig. 5E and F). Surprisingly, the *virK4-mut-up-for* mutant template was transcribed as well as the wild-type template (Fig. 5E and F), which is in contrast to the defective transcription displayed *in vivo* (Fig. 5D). However, when H-NS was added to the reactions, the *virK4-mut-up-for* mutant template was not transcribed as efficiently as the wild type (Fig. 5G–J). These data suggest that transcriptional activation of the *virK* promoter requires binding of PhoP to one PhoP box to help overcome H-NS-mediated silencing and to another PhoP box to recruit RNAP.

It has been proposed that PhoP overcomes H-NS silencing at the *virK* promoter by binding to a PhoP box that is different from the two PhoP boxes described above (Zhao *et al*., 2008). To test this notion, we evaluated the fluorescence produced by a strain with an isogenic plasmid harbouring nucleotide substitutions in the proposed PhoP box (i.e. *virK4-mut-up-rev*; Fig. 5A). The resulting strain produced similar fluorescence as the strain with the wild-type *virK* promoter (Fig. 5E). This *in vivo* result suggests that the proposed PhoP box is neither necessary to activate the *virK* promoter nor necessary to overcome silencing by H-NS.

### Promoter architectures differ between ancestral and horizontally acquired genes

**Fig. 6:**
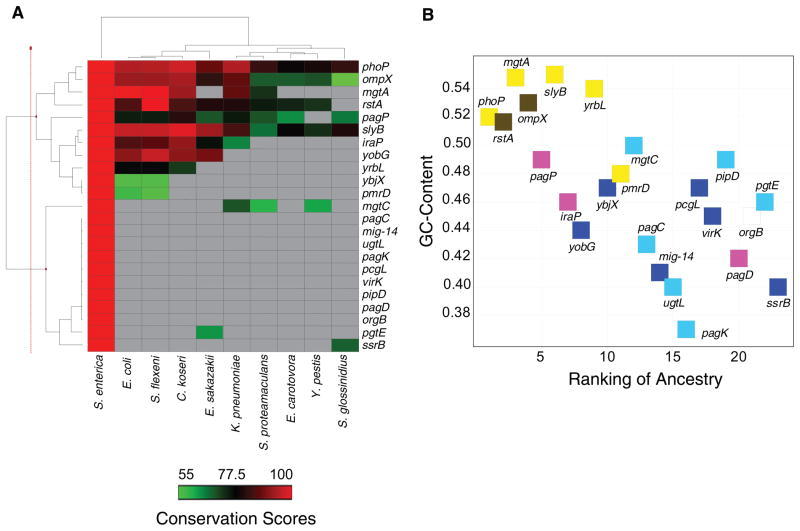
Relation between promoter architecture and the ancestral and horizontally acquired nature of PhoP-activated genes. A. Hierarchical clustering (Statistical Toolbox, Matlab 2007b, complete linkage method and correlation similarity were applied) of the Conservation Scores (CS) across 10 species of proteins encoded by 23 PhoP-activated genes in *Salmonella*. CS is calculated as in Janky and van Helden (2007) (*e*-value < 1E-5, expected number of false positives in a reciprocal blast search). CS values (represented by colours) can range from 55% to 100% when the closest homologue exhibits that percentage of amino acid identity (grey colour corresponds to CS < 55%). (Note that the phylogenetic relationships shown at the top of the figures do not represent phylogenetic distances.) Top cluster groups genes exhibiting the highest CSs among the evaluated bacterial genomes (*Salmonella enterica* serovar Typhimurium 14028s; *Shigella flexneri* 2a; *Escherichia coli* K12; *Citrobacter koseri* ATCC BAA-895; *Enterobacter sakazakii* ATCC BAA-894; *Klebsiella pneumoniae* MGH 78578; *Sodalis glossinidius morsitans*; *Erwinia carotovora atroseptica* SCRI1043; *Serratia proteamaculans* 568; and *Yersinia pestis* KIM). Bottom cluster groups genes exhibiting the lowest CSs along the evaluated bacterial genomes. B. Scatter plot combining the ranking of ancestry with the CG-content (Bioinformatics Toolbox, Matlab 2007b) of the 23 proteins in *Salmonella* (*F* statistic *P*-value < 0.0005). The ranking of ancestry is defined as the order in which genes were incorporated into the clusters plotted in (A) (i.e. order among conserved ORF sequences across species, see *Supporting information*). Colours correspond to promoter architectures (yellow: I, blue: II, brown: III, magenta: IV, cyan: V. The empty square indicates a reclassification of the *orgB* promoter).

We analysed the genes corresponding to the 23 PhoP-activated promoters investigated in this work to determine their ancestral versus horizontally acquired nature based on the Conservation Scores (CS) (Janky and van Helden, 2007) of the corresponding ORFs (Fig. 6A) and their respective GC contents (Ochman *et al*., 2000). The CS is a measure of the degree of sequence identity for each pair of homologues by calculating their reciprocal blast scores from a *Salmonella* protein and its closest homologue in a particular species. CS values range from 0 when no homologue or orthologue is detected in another species to 100% amino acid identity. Proteins with amino acid identity below the median ∼ 55% identity are unlikely to be true orthologues (Fig. 6A, grey colour). CSs are arranged in matrices where the *x*-axis corresponds to different species and *y*-axis corresponds to the *Salmonella* genes investigated in this study. Then, we grouped genes present in similar species (CS > 55%) and calculated the rank order in which genes were incorporated into the corresponding groups (i.e. order among conserved ORF sequences across species, see *Experimental procedures*) (Fig. 6A). The cross-correlation between this rank order and the GC content of the genes reveals a highly linear relation between them (*F* statistics, *P*-value < 0.0005, Fig. 6B). This model suggests that those genes with a rank order < 10 and GC content > 0.5 correspond to ancestral genes (Fig. 6B, top left). By contrast, genes with a rank order > 10 (Fig. 6B) and GC content < 0.5 correspond to horizontally acquired genes (Fig. 6B, bottom right).

We then examined whether ancestral and horizontally acquired genes harboured distinct promoter architectures. We determined that ancestral genes are primarily driven by promoters with architectures I and III (Figs 2 and 6B), whereas horizontally acquired genes are transcribed from promoters with architectures II, IV and V (Figs 2 and 6B). The *orgB* promoter with architecture I (Figs 2 and 6B), rank order > 10 and GC content < 0.5 (Fig. 6B) constitutes an exception within the present data distribution. Because a leave-one-out analysis points to *orgB* as an outlier (see *Experimental procedures*), we investigated its regulatory region with position weight matrices resulting from the sequences footprinted by PhoP-P (Fig. 1 and Fig. S3). We identified a putative second PhoP box (i.e. CTGTTGAGGGGATACTGAT) resembling submotif S3 (Fig. 1C), in the direct orientation and located 56 nt upstream of the transcription start site, which is similar to the location of PhoP boxes in the *pipD* and *mgtC* promoters (Table 1). Therefore, *orgB* appears to be driven by an architecture V promoter. Our analysis shows that the promoters of PhoP-activated ancestral genes typically have simpler architecture (e.g. a single PhoP box at discrete locations), whereas those activating transcription of horizontally acquired genes are more varied and complex (Fig. 6B, see colour distributions along the linear model).

## Discussion

The PhoP-P protein utilizes a variety of promoter architectures to activate gene transcription in *S. enterica* serovar Typhimurium. Our analysis is based on the experimentally validated *cis*-acting features of 23 PhoP-activated natural promoters and mutational analysis of these features. Therefore, it differs from approaches that focused on a single promoter or that used artificial promoters (Mayo *et al*., 2006; Lu *et al*., 2009), which has led others to propose forms of PhoP activation that either were incomplete (Lejona *et al*., 2003) or could not be reconciled with our present understanding of activators functioning with the σ^70^ form of RNAP (Gal-Mor *et al*., 2011). We propose that the architectures identified in this work are critical contributors to the regulatory programme carried out by the PhoP protein.

The main conclusions of our investigations are as follows: first, PhoP-P uses only particular combinations of *cis*-regulatory features (i.e. promoter architectures) to activate transcription from its promoters. This is in contrast to the suggestion that PhoP-activated promoters can ‘tolerate’ different orientations and locations of the PhoP box(es) (Zhao *et al*., 2008). In fact, we demonstrated that specific, non-arbitrary combinations of *cis*-acting sequences comprise these architectures because: (i) each class of architecture is shared by multiple promoters, indicating that each architecture was adopted due to functional constraints, and (ii) only a few of the potential arrangements of the *cis-*acting features are found. Unlike regulators such as the cAMP receptor (CRP) (Barnard *et al*., 2004; Browning and Busby, 2004), PhoP seems to promote transcription not only from promoters having PhoP boxes located at integral turns away from the predicted −10 hexamer (e.g. Architecture III promoters; Fig. 2), but also from promoters with PhoP boxes in the reverse orientation and located at half-integral turns away from the predicted −10 hexamer (e.g. Architecture V promoters; Fig. 2). In addition, we established that the PhoP box can function from more than one location up to a certain distance from the predicted −10 hexamer, and spaced by integral and half-integral turns of the DNA helix from this sequence (Fig. 4C and D). At any given position, the PhoP box is active only in one of the two possible orientations as the PhoP box conferred high levels of expression when present 48 nt upstream of the predicted −10 hexamer in the reverse orientation but no expression when in the direct orientation at the same location (Fig. 4C and D). The converse was true when the PhoP box was present 12 nt upstream of the predicted −10 hexamer sequence (Fig. 4C and D).

All 23 experimentally verified PhoP-activated promoters have at least one PhoP box upstream of the predicted −10 hexamer (Table 1; Fig. 2). Yet, it has been proposed that the PhoP-activated *sseL* gene is driven by a promoter harbouring two PhoP boxes: one overlapping the transcription start site and the other one downstream of the start site (Gal-Mor *et al*., 2011). Thus, it is not clear how PhoP can initiate transcription from the *sseL* promoter rather than interfere with RNAP binding and/or transcription elongation. Moreover, we could not identify a single instance of a promoter harbouring a PhoP box in the reverse orientation, located < 24 nt upstream of the predicted −10 hexamer, and separated by half-integral turns of the DNA helix from that hexamer in the 23 PhoP-activated promoters analysed in this study (Fig. 2).

None of the natural PhoP-activated promoters described to date harbours a single PhoP box > 30 nt upstream of the predicted −10 hexamer, in the direct orientation and/or separated by integral turns of the DNA helix from that hexamer (Fig. 2). Yet, others have proposed an unusual promoter architecture for several PhoP-activated genes (Zhao *et al*., 2008). For instance, the *pagD* promoter was proposed to have a single PhoP box in the direct orientation located 144 nt away from the transcription start site (Zhao *et al*., 2008). Thus, it is unclear how PhoP-P would interact with RNAP from the proposed location. By contrast, our investigations revealed the presence of a second PhoP box in the *pagD* promoter, one that is footprinted by PhoP-P. The identified PhoP box is present in the direct orientation 22 nt upstream of the predicted −10 hexamer (Table 1; Fig. 2). The different promoter architectures driving transcription of the *pagD* (Architecture IV; Fig. 2) and the divergent *pagC* (Architecture V; Fig. 2) genes suggest they are likely to be independently activated by the PhoP protein [as opposed to them being co-ordinately transcribed by PhoP-P binding to a single PhoP box as suggested by others (Zhao *et al*., 2008)].

Second, promoter architectures are associated with the ancestral or horizontally acquired character of the corresponding genes rather than the function of the encoded gene products. For instance, both the ancestral *mgtA* gene and the horizontally acquired *mgtB* gene specify Mg^2+^ transporters (Groisman, 2001), yet, they are transcribed from promoters with different architectures (Fig. 2).

Third, the length of the 5′ leader regions of PhoP-activated transcripts, which can be quite long, is not correlated with promoter architecture (Fig. S2B) or gene ancestry (Fig. S2C). For example, the *mgtA* and the *mgtC* transcripts have similarly long leader regions, but differ both in the architectures of their respective promoters and in their gene ancestries (Table 1; Figs 2 and 6; and Fig. S2B and C).

Fourth, PhoP-P plays at least four distinct roles when promoting transcription directly. For architecture I and III promoters, which have the PhoP box overlapping and upstream of the proposed −35 hexamer, respectively, PhoP-P is predicted to establish interactions with different RNAP subunits (Fig. 3) (Perez and Groisman, 2009). Most transcription factors activate transcription by making contact with either the α-CTD or the σ^70^ subunit of RNAP (Hochschild and Dove, 1998). The PhoP protein appears to interact with the α-CTD in promoter architectures having a PhoP box that does not overlap with the −35 hexamer. In addition, PhoP, like the PhoB and VanR proteins (Blanco *et al*., 2002; Depardieu *et al*., 2005), is predicted to contact the σ subunit of RNAP in promoter architectures having a PhoP box completely overlapping the −35 hexamer. Unlike the λ cII protein, which requires the α-CTD when cII-specific direct repeats straddle the promoter −35 element (Jain *et al*., 2005), the PhoP protein does not require contacting the α-CTD when the PhoP box overlaps the −35 in the *mgtA* promoter (Yamamoto *et al*., 2002; Perez and Groisman, 2009). Moreover, like the CRP protein (Salles and Weinstock, 1989; Kolb *et al*., 1993; Busby and Ebright, 1999; Tebbutt *et al*., 2002), PhoP activates promoters harbouring more than one binding site located upstream from the predicted −10 hexamer, which may require PhoP-P to interact with different RNAP subunits at the same promoter. This suggests that the PhoP protein can interact with RNAP in multiple ways due to the varied architectures of its promoters.

In addition, PhoP can promote transcription via mechanisms that do not involve interactions with RNAP. For instance, PhoP also activates transcription from the *ugd* promoter in a manner dependent on the response regulator RcsB (Mouslim *et al*., 2003). PhoP binds to a single site located 145 nt upstream from the RcsB-dependent transcription start site where it may interact with RcsB and/or alter the *ugd* promoter in a way that aids RcsB binding and/or recruitment of RNAP (Mouslim *et al*., 2003).

And fifth, promoter architectures correlate relatively well (Table 1, *F* statistics, *P*-value < 0.01) with promoter strength as measured by expression microarray analysis of wild-type versus phoP mutant strains (Harari *et al*., 2010). The *yrbL*, *pmrD* and *mgtA* genes are an exception to this correlation. This could reflect that the *pmrD* and *yrbL* promoters are directly repressed by the response regulator PmrA, which is activated post-translationally by the PhoP-dependent PmrD protein (Kato *et al*., 2003; Zwir *et al*., 2005; Harari *et al*., 2010). And in the case of *mgtA*, the transcript levels depend on its 5′ leader region (Cromie *et al*., 2006; Park *et al*., 2011).

In those PhoP-activated promoters that harbour two PhoP boxes, one of the PhoP boxes is required for activation whereas the second PhoP box plays a variety of functions depending on its location. PhoP boxes located upstream of the predicted −10 proximal PhoP box stimulate gene transcription (Fig. 5C). In the case of the *ybjX* promoter, mutation of the PhoP box present at the site normally corresponding to the −35 hexamer abolishes transcription whereas mutation of the second PhoP box, which is located upstream of the first PhoP box, decreases but does not eliminate transcription (Fig. 5B). Like the architecture II *ybjX* promoter (Fig. 2), the *virK* promoter harbours two PhoP boxes: one where the −35 hexamer is normally present, which is also required for transcription (Fig. 5D), and one further upstream that serves to overcome H-NS-mediated silencing (Fig. 5D).

Promoters with architecture V also harbour at least two PhoP boxes (Fig. 2). The location of the first PhoP box corresponds to an activation site (Fig. 2), whereas the location of the second PhoP box – either overlapping the predicted −10 hexamer or downstream of the transcription start site (Fig. 2) – is anticipated to repress or silence gene expression. Our data suggest that PhoP-P, apparently acting on its own, binds at one site to activate transcription and at another site to repress transcription in the same promoter (Fig. S4) when *Salmonella* experiences inducing conditions for the PhoP/PhoQ system. This distinguishes PhoP-P from other forms of regulation where distinct activators and repressors bind to different sites in the same promoter (Valentin-Hansen *et al*., 1996; Roy *et al*., 1998; Browning *et al*., 2000; 2002; Barnard *et al*., 2004; Guido *et al*., 2006), or where the same protein can function as an activator in one context and as a repressor in another (Ansari *et al*., 1995; Li *et al*., 1997; Roy *et al*., 1998; 2004).

The PhoP-P protein has less affinity for the second PhoP box than for the first PhoP box (Fig. S3), suggesting that the PhoP-P protein occupies the second PhoP box after the first one. This arrangement could give rise to an expression behaviour whereby a gene is expressed only when *Salmonella* experiences a discrete range of inducing conditions that produce intermediate levels of PhoP-P. This is because at high PhoP-P concentrations, PhoP-P would bind to the second PhoP box, silencing expression. Therefore, as PhoP-P positively regulates its own transcription (Soncini *et al*., 1995), which results in a continuous increase of PhoP-P levels during the first 30 min of the bacterium experiencing inducing conditions (Shin *et al*., 2006), promoters having two PhoP boxes, one of which located downstream of or overlapping the predicted −10 region, may give rise to a time-dependent expression over a particular time period. The proposed scenario is analogous to that previously demonstrated for the *bipA* gene from *Bordetella bronchiseptica*, which is expressed at intermediate activation levels for the BvgA/BvgS regulatory system (Stockbauer *et al*., 2001), and is controlled by a promoter with a high-affinity BvgA binding site involved in activation and a low-affinity binding site involved in repression (Williams *et al*., 2005).

PhoP-activated promoters can also harbour sites for other DNA-binding proteins. For example, the SlyA protein binds the *pagC* and *ugtL* promoters to overcome silencing by the H-NS protein (Perez *et al*., 2008). In agreement with previous results (Kong *et al*., 2008), we showed that the PhoP protein, like the SlyA protein (Perez *et al*., 2008), can function to overcome silencing by H-NS by using a second PhoP box such as that of the H-NS silenced *virK* promoter. It has been proposed that the role of the PhoP protein at these complex promoters is limited to antagonizing H-NS (Kong *et al*., 2008; Zhao *et al*., 2008). However, we demonstrated that the PhoP protein is required to promote activation from the *pagC* promoter *in vitro*, even when H-NS was not included in the reaction (Perez *et al*., 2008). In addition, the PmrA protein binds the *pmrD* promoter (Kato *et al*., 2003) to repress its transcription, and it is predicted to bind and repress the *Salmonella yrbL* promoter as it does for the *E. coli yrbL* promoter (Zwir *et al*., 2005). The *ugd* promoter has binding sites for the PmrA, PhoP and RcsB proteins, and the latter two proteins are necessary to promote *ugd* transcription under certain inducing conditions (Mouslim and Groisman, 2003).

It has been postulated that the PhoP and SlyA proteins control expression of a group of divergently transcribed genes (Zhao *et al*., 2008). Because the proposed location of the PhoP boxes was excessively far upstream from the mapped transcription start sites, the authors proposed that the PhoP box was acting to overcome H-NS rather than to recruit RNAP in these promoters (Zhao *et al*., 2008). However, this does not appear to be the case, at least for some of the proposed genes, because we could identify other PhoP boxes at locations matching those of known promoter architectures (Fig. 2). For example, we demonstrated that the PhoP box identified in the *virK* and *pipB2* promoter region (Zhao *et al*., 2008) (*virK-mut-up-rev*; Fig. 5D), located 55 nt and 121 nt upstream of the predicted −10 hexamer in the *virK* and *pipB2*, respectively, has no effect on the *virK* transcription (Fig. 5D). By contrast, a PhoP box located 12 nt upstream of the predicted −10 hexamer is required for *virK* transcription (Fig. 5D). Likewise, the location of the PhoP box proposed for the *pipB2* promoter (Zhao *et al*., 2008) is too far away from the RNAP binding site to be functional. Indeed, we identified a PhoP box in the direct orientation located ∼ 20 nt upstream of the predicted −10 hexamer (Harari *et al*., 2010) like that present in the *rstA* and *ompX* promoters with architecture III (Fig. 2). This may be the case of the STM1632 promoter, where we identified a PhoP box in the direct orientation and located 13 nt upstream of the predicted −10 hexamer, which is typical of architecture I promoters and in contrast to the proposal of others (Zhao *et al*., 2008). In addition, PhoP boxes consistent with promoter architecture V have been identified in the *pepN*, *STM2585* (Harari *et al*., 2010), STM3124 and *ppiA* promoters, located 59 nt, 48 nt, 47 nt and 36 nt upstream of the predicted −10 sequence respectively.

Finally, certain promoter architectures appear to be species specific. For instance, the PhoP-activated *mgtC* promoter from *Yersinia pestis* harbours a PhoP box at a location/orientation that has yet to be identified among PhoP-activated promoters in *Salmonella*, and similarly, the architecture of the *Salmonella ugtL* promoter has not been encountered in the *Yersinia* PhoP regulon (Perez *et al*., 2008; Perez and Groisman, 2009). Notably, the PhoP proteins from *Salmonella* and *Yersinia* have diverged, and the *Salmonella* protein is unable to promote transcription from the *Yersinia mgtC* promoter; yet, it can bind to this promoter just like the *Yersinia* PhoP protein and promote transcription from other promoters having conserved architectures (Perez *et al*., 2008; Perez and Groisman, 2009). Likewise, the *Yersinia* PhoP protein could not transcribe the *Salmonella ugtL* promoter (Perez *et al*., 2008; Perez and Groisman, 2009). This suggests that caution must be exercised when trying to deduce whether a gene is under the control of a given regulator because a binding site for such regulator is present in the identified promoter region.

## Experimental procedures

### DNase I footprinting assays

Primers A2, B2, C2, D2, E2, F2, G2, H2, I2, J2, K2, L2, M2 and N2 (Table S1) were labelled with 2 units of T4 polynucleotide kinase and 10 pmol of [γ-^32^P]-ATP using 5 µl of 5 × forward buffer (Gibco/BRL) in a total volume of 25 µl at 37° C for 1 and 2 h and unincorporated ^32^P was removed using ProbeQuant G-50 microcolumns (GE Healthcare). Then, the labelled primer and its partner unlabelled primer (A1 and A2, B1 and B2, C1 and C2, D1 and D2, E1 and E2, F1 and F2, G1 and G2, H1 and H2, I1 and I2, J1 and J2, K1 and K2, L1 and L2, M1 and M2) were used to generate corresponding PCR fragments using genomic DNA from strain 14028s. DNase I footprinting assays were carried out as described in Kato *et al*. (2003) using different concentrations of PhoP-P-His6 and 0.02 units of DNase I (Epicentre).

### SI nuclease-protection assay

SI nuclease-protection assays were performed as described (Kox *et al*., 2000) with RNA harvested from early exponential (OD_600_, 0.3–0.4) phase cultures of wild-type (14028s) and *phoP* (MS7953s) *Salmonella* grown in 30 ml of N-minimal medium, pH 7.4, containing either 10 mM MgCl_2_ or 10 µM MgCl_2_. Total RNA was isolated using SV Total RNA Isolation System (Promega, Madison, WI) according to the manufacturer's specifications. The RNA was hybridized to fragments generated by PCR using chromosomal DNA was a substrate. Probes used for *mig-14*, *pagK*, *yobG*, *pagP*, *ybjX*, *slyB*, *pcgL*, *pagD* and *virK* were the same as those used for DNase I footprinting (Table S1). Probes for *yrbL*, *ompX*, *pipD* and *mgtC* were generated by labelling C2, J4, K3, M4 and using PCR as described above with labelled and unlabelled primers C3 and C2, J3 and J4, K1 and K3 and M3 and M4 respectively (Table S2).

### Bacterial strains, plasmids and growth conditions

Bacterial strains, plasmids, and primers used in the GFP assay are listed in Table S3 and S4. *S. enterica* serovar Typhimurium strains used in this study were derived from strain 14028s. Bacteria were grown at 37°C with aeration in N-minimal media (pH 7.7), supplemented with 0.1% casamino acids, 38 mM glycerol and 10 µM or 10 mM MgCl_2_.

### Construction of plasmids harbouring fusions between PhoP-activated promoters and a promoterless *gfp* gene

Plasmid pMS-*virK*, encoding the *virK* promoter region fused to a promoterless *gfp* gene, was constructed by cloning a PCR fragment generated using primers 9224 and 9225 between the BamHI and XhoI sites of plasmid pMS201 (Mangan and Alon, 2003).

Plasmid pMS-*virK*-mut-up-for, encoding the *virK* promoter region with the mutated upstream PhoP box in the direct orientation fused to a promoterless *gfp* gene, was constructed by cloning a PCR fragment generated using primers 9228 and 9229 between the BamHI and XhoI sites of plasmid pMS201 (Mangan and Alon, 2003).

Plasmid pMS-*virK*-mut-up-rev, encoding the *virK* promoter region with the mutated upstream PhoP box in the reverse orientation fused to a promoterless *gfp* gene, was constructed by cloning a PCR fragment generated using primers 9226 and 9227 between the BamHI and XhoI sites of plasmid pMS201 (Mangan and Alon, 2003).

Plasmid pMS-*virK*-mut-down, encoding the *virK* promoter region with the mutated downstream PhoP box fused to a promoterless *gfp* gene, was constructed by cloning a PCR fragment generated using primers 9231 and 9230 between the BamHI and XhoI sites of plasmid pMS201 (Mangan and Alon, 2003).

Plasmid pMS-*phoP4*, encoding a shortened derivative of the *phoP* promoter region including the PhoP box and the predicted binding site fused to a promoterless *gfp* gene, was constructed by cloning a PCR fragment generated using primers 8142 and 8143 between the BamHI and XhoI sites of plasmid pMS201 (Mangan and Alon, 2003).

Plasmid pMS-*phoP4*-rev, encoding the *phoP4* promoter region where the PhoP box in the direct orientation was placed in the opposite orientation and fused to a promoterless *gfp* gene, was constructed by cloning a PCR fragment generated using primers 8233 and 8234 between the BamHI and XhoI sites of plasmid pMS201 (Mangan and Alon, 2003).

Plasmid pMS-*rstA4*, encoding a shortened derivative of the *rstA* promoter region including the PhoP box and the predicted binding site fused to a promoterless *gfp* gene, was constructed by cloning a PCR fragment generated using primers 8144 and 8145 between the BamHI and XhoI sites of plasmid pMS201 (Mangan and Alon, 2003).

Plasmid pMS-*rstA4*-rev, encoding the *rstA4* promoter region where the PhoP box in the direct orientation was placed in the opposite orientation and fused to a promoterless *gfp* gene, was constructed by cloning a PCR fragment generated using primers 8235 and 8236 between the BamHI and XhoI sites of plasmid pMS201 (Mangan and Alon, 2003).

Plasmid pMS-*phoP4*-rev-K1, encoding the *phoP4* promoter region where the PhoP box was placed in the opposite orientation and moved further upstream at the position normally occupied by the PhoP box in the *pagK* promoter and fused to a promoterless *gfp* gene, was constructed by cloning a PCR fragment generated using primers 8604 and 8592 between the BamHI and XhoI sites of plasmid pMS201 (Mangan and Alon, 2003).

Plasmid pMS-*phoP4*-rev-C, encoding the *phoP4* promoter region where the PhoP box was placed in the opposite orientation and moved further upstream at the position normally occupied by the PhoP box in the *mgtC* promoter and fused to a promoterless *gfp* gene, was constructed by cloning a PCR fragment generated using primers 8489 and 8490 between the BamHI and XhoI sites of plasmid pMS201 (Mangan and Alon, 2003).

Plasmid pMS-*phoP*, encoding the *phoP* promoter region fused to a promoterless *gfp* gene, was constructed by cloning a PCR fragment generated using primers 4811 and 4432 between the BamHI and XhoI sites of plasmid pMS201 (Mangan and Alon, 2003).

Plasmid pMS-*rstA*, encoding the *rstA* promoter region fused to a promoterless *gfp* gene, was constructed by cloning a PCR fragment generated using primers 4842 and 5231 between the BamHI and XhoI sites of plasmid pMS201 (Mangan and Alon, 2003).

Plasmid pMS-*phoP4-*7nt, encoding the *phoP4* promoter region where the PhoP box was located 7 nt upstream of the predicted −10 hexamer and fused to a promoterless *gfp* gene, was constructed by cloning a PCR fragment generated using primers 8694 and 8693 between the BamHI and XhoI sites of plasmid pMS201 (Mangan and Alon, 2003).

Plasmid pMS-*phoP4-*17nt, encoding the *phoP4* promoter region where the PhoP box was located 17 nt upstream of the predicted −10 hexamer and fused to a promoterless *gfp* gene, was constructed by cloning a PCR fragment generated using primers 8691 and 8692 between the BamHI and XhoI sites of plasmid pMS201 (Mangan and Alon, 2003).

Plasmid pMS-*phoP4-*12nt, encoding the *phoP4* promoter region where the PhoP box was located 12 nt upstream of the predicted −10 hexamer and fused to a promoterless *gfp* gene, was constructed by cloning a PCR fragment generated using primers 8142 and 8143 between the BamHI and XhoI sites of plasmid pMS201 (Mangan and Alon, 2003).

Plasmid pMS-*phoP4-*21nt, encoding the *phoP4* promoter region where the PhoP box was located 21 nt upstream of the predicted −10 hexamer and fused to a promoterless *gfp* gene, was constructed by cloning a PCR fragment generated using primers 8736 and 8749 between the BamHI and XhoI sites of plasmid pMS201 (Mangan and Alon, 2003).

Plasmid pMS-*phoP4-*22nt, encoding the *phoP4* promoter region where the PhoP box was located 22 nt upstream of the predicted −10 hexamer and fused to a promoterless *gfp* gene, was constructed by cloning a PCR fragment generated using primers 8737 and 8750 between the BamHI and XhoI sites of plasmid pMS201 (Mangan and Alon, 2003).

Plasmid pMS-*phoP4-*23nt, encoding the *phoP4* promoter region where the PhoP box was located 23 nt upstream of the predicted −10 hexamer and fused to a promoterless *gfp* gene, was constructed by cloning a PCR fragment generated using primers 8545 and 8548 between the BamHI and XhoI sites of plasmid pMS201 (Mangan and Alon, 2003).

Plasmid pMS-*phoP4-*24nt, encoding the *phoP4* promoter region where the PhoP box was located 24 nt upstream of the predicted −10 hexamer and fused to a promoterless *gfp* gene, was constructed by cloning a PCR fragment generated using primers 8738 and 8751 between the BamHI and XhoI sites of plasmid pMS201 (Mangan and Alon, 2003).

Plasmid pMS-*phoP4-*25nt, encoding the *phoP4* promoter region where the PhoP box was located 25 nt upstream of the predicted −10 hexamer and fused to a promoterless *gfp* gene, was constructed by cloning a PCR fragment generated using primers 8739 and 8752 between the BamHI and XhoI sites of plasmid pMS201 (Mangan and Alon, 2003).

Plasmid pMS-*phoP4-*26nt, encoding the *phoP4* promoter region where the PhoP box was located 26 nt upstream of the predicted −10 hexamer and fused to a promoterless *gfp* gene, was constructed by cloning a PCR fragment generated using primers 8740 and 8753 between the BamHI and XhoI sites of plasmid pMS201 (Mangan and Alon, 2003).

Plasmid pMS-*phoP4-*27nt, encoding the *phoP4* promoter region where the PhoP box was located 27 nt upstream of the predicted −10 hexamer and fused to a promoterless *gfp* gene, was constructed by cloning a PCR fragment generated using primers 8741 and 8754 between the BamHI and XhoI sites of plasmid pMS201 (Mangan and Alon, 2003).

Plasmid pMS-*phoP4-*28nt, encoding the *phoP4* promoter region where the PhoP box was located 28 nt upstream of the predicted −10 hexamer and fused to a promoterless *gfp* gene, was constructed by cloning a PCR fragment generated using primers 8613 and 8593 between the BamHI and XhoI sites of plasmid pMS201 (Mangan and Alon, 2003).

Plasmid pMS-*phoP4-*33nt, encoding the *phoP4* promoter region where the PhoP box was located 33 nt upstream of the predicted −10 hexamer and fused to a promoterless *gfp* gene, was constructed by cloning a PCR fragment generated using primers 8597 and 8602 between the BamHI and XhoI sites of plasmid pMS201 (Mangan and Alon, 2003).

Plasmid pMS-*phoP4-*48nt, encoding the *phoP4* promoter region where the PhoP box was located 48 nt upstream of the predicted −10 hexamer and fused to a promoterless *gfp* gene, was constructed by cloning a PCR fragment generated using primers 8487 and 8488 between the BamHI and XhoI sites of plasmid pMS201 (Mangan and Alon, 2003).

Plasmid pMS-*phoP4*-rev-28nt, encoding the *phoP4* promoter region where the PhoP box in the opposite orientation was located 28 nt upstream of the predicted −10 hexamer and fused to a promoterless *gfp* gene, was constructed by cloning a PCR fragment generated using primers 8732 and 8745 between the BamHI and XhoI sites of plasmid pMS201 (Mangan and Alon, 2003).

Plasmid pMS-*phoP4*-rev-29nt, encoding the *phoP4* promoter region where the PhoP box in the opposite orientation was located 29 nt upstream of the predicted −10 hexamer and fused to a promoterless *gfp* gene, was constructed by cloning a PCR fragment generated using primers 8731 and 8744 between the BamHI and XhoI sites of plasmid pMS201 (Mangan and Alon, 2003).

Plasmid pMS-*phoP4*-rev-30nt, encoding the *phoP4* promoter region where the PhoP box in the opposite orientation was located 30 nt upstream of the predicted −10 hexamer and fused to a promoterless *gfp* gene, was constructed by cloning a PCR fragment generated using primers 8730 and 8743 between the BamHI and XhoI sites of plasmid pMS201 (Mangan and Alon, 2003).

Plasmid pMS-*phoP4*-rev-31nt, encoding the *phoP4* promoter region where the PhoP box in the opposite orientation was located 31 nt upstream of the predicted −10 hexamer and fused to a promoterless *gfp* gene, was constructed by cloning a PCR fragment generated using primers 8729 and 8742 between the BamHI and XhoI sites of plasmid pMS201 (Mangan and Alon, 2003).

Plasmid pMS-*phoP4*-rev-36nt, encoding the *phoP4* promoter region where the PhoP box in the opposite orientation was located 36 nt upstream of the predicted −10 hexamer and fused to a promoterless *gfp* gene, was constructed by cloning a PCR fragment generated using primers 8441 and 8438 between the BamHI and XhoI sites of plasmid pMS201 (Mangan and Alon, 2003).

Plasmid pMS-*phoP4*-rev-47nt, encoding the *phoP4* promoter region where the PhoP box in the opposite orientation was located 47 nt upstream of the predicted −10 hexamer and fused to a promoterless *gfp* gene, was constructed by cloning a PCR fragment generated using primers 8734 and 8747 between the BamHI and XhoI sites of plasmid pMS201 (Mangan and Alon, 2003).

Plasmid pMS-*phoP4*-rev-26nt, encoding the *phoP4* promoter region where the PhoP box in the opposite orientation was located 26 nt upstream of the predicted −10 hexamer and fused to a promoterless *gfp* gene, was constructed by cloning a PCR fragment generated using primers 8733 and 8746 between the BamHI and XhoI sites of plasmid pMS201 (Mangan and Alon, 2003).

Plasmid pMS-*phoP4*-rev-27nt, encoding the *phoP4* promoter region where the PhoP box in the opposite orientation was located 27 nt upstream of the predicted −10 hexamer and fused to a promoterless *gfp* gene, was constructed by cloning a PCR fragment generated using primers 8608 and 8601 between the BamHI and XhoI sites of plasmid pMS201 (Mangan and Alon, 2003).

Plasmid pMS-*phoP4*-rev-32nt, encoding the *phoP4* promoter region where the PhoP box in the opposite orientation was located 32 nt upstream of the predicted −10 hexamer and fused to a promoterless *gfp* gene, was constructed by cloning a PCR fragment generated using primers 8598 and 8606 between the BamHI and XhoI sites of plasmid pMS201 (Mangan and Alon, 2003).

Plasmid pMS-*phoP4*-rev-37nt, encoding the *phoP4* promoter region where the PhoP box in the opposite orientation was located 37 nt upstream of the predicted −10 hexamer and fused to a promoterless *gfp* gene, was constructed by cloning a PCR fragment generated using primers 8604 and 8592 between the BamHI and XhoI sites of plasmid pMS201 (Mangan and Alon, 2003).

Plasmid pMS-*phoP4*-rev-48nt, encoding the *phoP4* promoter region where the PhoP box in the opposite orientation was located 48 nt upstream of the predicted −10 hexamer and fused to a promoterless *gfp* gene, was constructed by cloning a PCR fragment generated using primers 8489 and 8490 between the BamHI and XhoI sites of plasmid pMS201 (Mangan and Alon, 2003).

Plasmid pMS-*ybjX*, encoding the *ybjX* promoter region fused to a promoterless *gfp* gene, was constructed by cloning a PCR fragment generated using primers 9253 and 9255 between the BamHI and XhoI sites of plasmid pMS201 (Mangan and Alon, 2003).

Plasmid pMS-*ybjX*-mut-up, encoding the *ybjX* promoter region with the mutated upstream PhoP box in the reverse orientation fused to a promoterless *gfp* gene, was constructed by cloning a PCR fragment generated using primers 9272 and 9273 between the BamHI and XhoI sites of plasmid pMS201 (Mangan and Alon, 2003).

Plasmid pMS-*ybjX*-mut-down, encoding the *ybjX* promoter region with the mutated downstream PhoP box fused to a promoterless *gfp* gene, was constructed by cloning a PCR fragment generated using primers 9293 and 9295 between the BamHI and XhoI sites of plasmid pMS201 (Mangan and Alon, 2003).

Plasmid pMS-*virK*-*phoP4*, encoding a shortened derivative of the *phoP* promoter region including the PhoP box and the predicted binding site fused to a promoterless *gfp* gene, was constructed by cloning a PCR fragment generated using primers 9436 and 9437 between the BamHI and XhoI sites of plasmid pMS201 (Mangan and Alon, 2003).

Plasmid pMS-*phoP4*-rev-23nt, encoding the *rstA4* promoter region where the PhoP box in the opposite orientation was located 23 nt upstream of the predicted −10 hexamer and fused to a promoterless *gfp* gene, was constructed by cloning a PCR fragment generated using primers 8235 and 8236 between the BamHI and XhoI sites of plasmid pMS201 (Mangan and Alon, 2003).

Plasmid pMS-*phoP4*-rev-K1-TTTAAT, encoding the *phoP4* promoter region where the PhoP box was placed in the opposite orientation and moved further upstream at the position normally occupied by the PhoP box in the *pagK* promoter and the original predicted −10 sequence was replaced by the ‘TTTAAT’ sequence and fused to a promoterless *gfp* gene, was constructed by cloning a PCR fragment generated using primers 9183 and 9184 between the BamHI and XhoI sites of plasmid pMS201 (Mangan and Alon, 2003).

Plasmid pMS-*phoP4*-rev-K1-TAATAT, encoding the *phoP4* promoter region where the PhoP box was placed in the opposite orientation and moved further upstream at the position normally occupied by the PhoP box in the *pagK* promoter and the original predicted −10 sequence was replaced by the ‘TAATAT’ sequence and fused to a promoterless *gfp* gene, was constructed by cloning a PCR fragment generated using primers 9189 and 9186 between the BamHI and XhoI sites of plasmid pMS201 (Mangan and Alon, 2003).

Plasmid pMS-*phoP4*-rev-C-TATAAT, encoding the *phoP4* promoter region where the PhoP box was placed in the opposite orientation and moved further upstream at the position normally occupied by the PhoP box in the *mgtC* promoter and the original predicted −10 sequence was replaced by the ‘TATAAT’ sequence and fused to a promoterless *gfp* gene, was constructed by cloning a PCR fragment generated using primers 8521 and 8524 between the BamHI and XhoI sites of plasmid pMS201 (Mangan and Alon, 2003).

Plasmid pMS-*phoP4*-rev-K1-TTGACA, encoding the *phoP4* promoter region where the PhoP box was placed in the opposite orientation and moved further upstream at the position normally occupied by the PhoP box in the *pagK* promoter and the original predicted −35 sequence was replaced by the ‘TTGACA’ sequence and fused to a promoterless *gfp* gene, was constructed by cloning a PCR fragment generated using primers 8607 and 8610 between the BamHI and XhoI sites of plasmid pMS201 (Mangan and Alon, 2003).

Plasmid pMS-*phoP4*+11nt-TATGTT, encoding the *phoP4* promoter region where the PhoP box was located 23 nt upstream of the predicted −10 hexamer and replaced by the ‘TATGTT’ sequence and fused to a promoterless *gfp* gene, was constructed by cloning a PCR fragment generated using primers 9181 and 9188 between the BamHI and XhoI sites of plasmid pMS201 (Mangan and Alon, 2003).

DNA sequencing was used to verify that the promoter regions had the predicted sequences.

### GFP reporter assay

Single colonies were used to inoculate 2 ml of cultures and grown for 16 h in N-minimal media at pH 7.7 (Snavely *et al*., 1991) with 10 mM Mg^2+^ and kanamycin (25 µg ml^−1^) at 37°C in a shaking incubator. The cultures were diluted 1:50 into 2 ml of the same media described above and grown an additional 4 h at 37°C in a shaking incubator. One millilitre of media cells was then harvested, washed once with 1 ml of N-minimal media containing 2.5 mM Mg^2+^ and resuspended in 200 µl of N-minimal media with 2.5 mM Mg^2+^. The concentrated cultures were diluted 1:50 in 150 µl of N-minimal media containing no Mg^2+^ or containing 10 mM Mg^2+^ in flat-bottomed 96-well plates (PerkinElmer 6005225, Waltham, MA). The final Mg^2+^ concentrations in these two conditions were 50 µM and 10 mM, which induce and repress the PhoP/PhoQ system respectively. The cultures were overlaid with 50 µl of mineral oil (Sigma M-3516, St. Louis, MO) to prevent evaporation. The GFP assay was performed as described (Kalir *et al*., 2001). The cultures were grown in a Wallac Victor^3^ multiwell fluorimeter (PerkinElmer) set at 37°C and assayed with an automatically repeating protocol of shaking (1 mm orbital, fast speed, 30 s), fluorescence readings (filters F485, F535, 0.5 s, CW lamp energy 10 000), and absorbance (OD) measurements (600 nm, P600 filter, 0.1 s) at 6 min intervals. Background fluorescence of cells bearing a promotorless GFP vector was subtracted from the values obtained with the cells harbouring plasmids with PhoP-activated promoters (or the engineered derivatives) fused to a promoterless *gfp* gene.

### GFP data pre-processing

The raw data corresponding to the GFP and OD signals were used to calculate the promoter activity as [d*Gi*(*t*)/d*t*]/*ODi*(*t*)] (Ronen *et al*., 2002). The activity signal, ODs and background were smoothed by a shape-preserving interpolant fitting algorithm (Piecewise Cubic Hermite Interpolating Polynomial, Matlab 7.1) that finds values of an underlying interpolating function at intermediate points not described in the experimental assays. Then, we applied a polynomial fit (sixth order) on each expression signal. This smoothing procedure captured the dynamic well, while removing the noise inherent in the differentiation of noisy signals (Ronen *et al*., 2002). Observed ODs were standardized (linear regressions *R*^2^ > 0.99) by using India ink from actual ODs measured by spectrophotometer. Observed fluorescence was standardized using dilutions of fluorescein and expressed as fluorescein concentrations.

### *In vitro* transcription assays

Linear DNA templates for *in vitro* transcription assays were generated by PCR using primer pair 7817 and 8302 and plasmid as a template: pMS-*virK*, pMS-*virK*-mut-up-for and pMS-*virK*-mut-down. *In vitro* single round transcription assays were performed as in Perez *et al*. (2008) and Perez and Groisman (2009). Briefly, a mixture of template DNA (0.15 pmol), purified His-tagged proteins, and RNA polymerase holoenzyme (Epicentre, Madison, WI) were incubated in 15 µl of transcription buffer [50 mM Tris-HCl (pH 8.0), 50 mM NaCl, 3 mM MgCl_2_, 0.1 mM EDTA, 0.1 mM DTT and 25 µg ml^−1^ BSA] for 30 min at 37°C to form open complexes. A 5 µl mixture of substrate and heparin was then added to make a final concentration of 160 µM each of ATP, GTP and CTP; 50 µM UTP; 2 µCi of [α-^32^P]-UTP; and 200 µg ml^−1^ heparin. After 10 min of incubation at 37°C, reactions were stopped by adding TBE-urea loading buffer (Invitrogen) and resolved in 10% TBE-Urea gels (Invitrogen). Overproduction and purification of proteins used in this assay were performed as in Perez *et al*. (2008) and Perez and Groisman (2009).

### Analysis of the PhoP submotifs and RNAP sequences

PhoP motifs were analysed as in Harari *et al*. (2010), and RNAP binding sites were identified based on methods described in Harari *et al*. (2009).

### Calculation of the ranking of ancestry

We applied a hierarchical agglomerative clustering (Statistical Toolbox, Matlab 2007b) with a complete linkage method and correlation similarity measurement to group both: gene products with similar CSs across 10 species (rows) as well as species harbouring similar gene products (columns). The function that controls the vertical order in which a row is plotted in the hierarchical clustering (Spotfire Decision Site 9.1.2) is defined as follows. Given two subclusters within a cluster (there are always exactly two subclusters), both subclusters are weighted and the subcluster with the highest weight is placed above the other subcluster. This function is systematically applied until a single cluster containing all rows is obtained. To calculate the weight *w*_3_ of a new cluster *C*_3_ formed from two subclusters *C*_1_ and *C*_2_ with a weight of *w*_1_ and *w*_2_, and each containing *n*_1_ and *n*_2_ rows, the following expression is used:


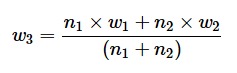


The weight of a subcluster with a single row is calculated as the average value of its columns.

### Correlation analysis

Correlations among submotifs and orientation, activation/repression PhoP boxes and promoter architectures have been performed calculating multiple linear regressions using the stepwise regression method, Statistics Toolbox, Matlab R2007b. Outlier identification has been performed using a univariate statistical approach, where the target distributions correspond to those of the correlation analysis and the outlier regions has been defined based on 0.95 confidence coefficient (Ben-Gal, 2005). A leave-one-out sampling process demonstrated that separating the *orgB* promoter improves the significances of the associations (*P*-values) ∼ 300%.
