# Supplementary material for: The promoter architectural landscape of the Salmonella PhoP regulon
Source: Mol Microbiol. 2012 Mar 27;84(3):463–85. doi: 10.1111/j.1365-2958.2012.08036.x (PMC3335776; doi:10.1111/j.1365-2958.2012.08036.x)
Supplement: Supplementary file 1 [file mmi0084-0463-SD1.pdf]

Supporting information

Supporting figure legends

Fig. S1. Mapping the transcription start site(s) of PhoP-activated promoters. S1 nuclease protection assay of the PhoP-activated *yrbL* (A), *ompX* (B), *yobG* (C), *pcgL* (D), *pagP* (E), *pagD* (F), *virK* (G), *mig-14* (H), *pagK* (I), *pgtE* (J), *mgtC* (K), *ybjX* (L) and *slyB* (M) promoters carried out with mRNAs harvested from wild-type (14028s) and *phoP* (MS7953s) *Salmonella* strains following growth in N-minimal medium with 10 mM (H) or 10 μM (L) Mg^{2+} concentrations. AG corresponds to Maxam-Gilbert A + G sequencing ladders. Arrows indicate the PhoP-dependent transcription start site, which are in green letters in Fig. 2.

Fig. S2. Analysis of the lengths of the 5′ leader regions corresponding to 23 PhoP-activated genes.

A. Histogram of the lengths of the 5′ leader regions.

B. Scatter plot combining the lengths of the 5′ leader regions of the promoters and their corresponding architectures (Fig. 2). Colors correspond to the ranking of ancestry as defined in Fig. 6 (see Experimental procedures). Ancestral and

horizontally-acquired genes are in red and green, respectively (The empty square indicates a reclassification of the *orgB* promoter).

C. Scatter plot combining the lengths of the 5′ leader regions of the promoters and their corresponding ranking of ancestry. Colors correspond to promoter architectures (yellow: I, blue: II, brown: III, magenta: IV, cyan: V. The empty square indicates a re-classification of the *orgB* promoter).

Fig. S3. The region protected by the PhoP-P protein in PhoP-activated promoters. DNase I footprinting analysis of promoter regions protected by the PhoP-P protein (black lines) at the *yrbL* (A), *slyB* (B), *ompX* (C), *rstA* (D), *pcgL* (E), *yobG* (F), *pagP* (G), *pagD* (H), *virK* (I), *mig-14* (J), *ybjX* (K), *pagK* (L), *pgtE* (M), *mgtC* (N), and *pipD* (O) promoters. The sequences protected by PhoP-P, as well as their corresponding locations, are indicated to the right of each gel (black lines). The concentrations of the purified PhoP used in each of the lanes in the gels are indicated at the top of each sub-figure.

Fig. S4. Identification of the predicted RNAP binding site in PhoP-activated promoters.

GFP-expression displayed by a *Salmonella* expressing HA-tagged PhoP from its normal chromosomal location (EG13918) harboring plasmids with a promoterless *gfp* gene driven by the *phoP4* promoter or derivatives with the indicated

subsitutions. Cells have been growth in N-minimal media containing 10 mM $MgCl_2$ and switched to mediat with 50 μM $MgCl_2$,. Promoter activity (*y*-axis) was measured by normalizing GFP expression to cell density and pre-proceeded as described in Experimental procedures, and the mean and S.D. values of at least three independent experiments are shown. The schematic represents the DNA (grey cylinder), where the PhoP box (blue cylinders) , the predicted -10 and -35 sequences (red cylinders), and the transcription start site are indicated.

A. The *phoP4+11* promoter (blue line) compared with its derivative (green line), where the predicted -10 region of *phoP4* was replaced by a weaker consensus sequence from the PhoP-activated *rstA* promoter.

B. The *phoP4+revK* promoter (blue line) compared with its derivative (green line), where the predicted -10 region of *phoP4* was replaced by weaker -10 consensus sequences.

C. The *phoP4+revC* promoter (blue line) compared with its derivative (green line), where the predicted -10 region of *phoP4* was replaced by a stronger -10 consensus sequence from the PhoP-activated *mgtC* promoter.

D. The *phoP4+revK* promoter (blue line) compared with its derivative (green line), where the predicted -35 region of *phoP4* was replaced by a stronger -35 consensus sequence.

Supporting tables

Table S1. Promoter-specific primers used in DNaseI footprinting and SI nuclease-protection assay

Number	Name	Sequence
6098	*A1: mig-14*	Forward 5′ GCGGCATTAAACCGTCGCTTTCAC 3′
6099	*A2: mig-14*	Reverse 5′ GCCGTATTGCGTAAACACCTC 3′
9199	*B1: pagK*	Forward 5′ GGATGGGAGCCAGTAAGGA 3′
9200	*B2: pagK*	Reverse 5′ CGCTCTTAACGTGTTTAATAATAC 3′
9220	*C1: yrbL*	Forward 5′ TAAACCTCCCCCTCTATAC 3′
4519	*C2: yrbL*	Reverse 5′ GTCACGGTTGTAAATGAC 3′
9205	*D1: yobG*	Forward 5′ CAGGGCAAACAATACAATTGCGC 3′
9204	*D2: yobG*	Reverse 5′ CGATGCCGAGAACGACCCATC 3′
5598	*E1: pagP*	Forward 5′ AAATCACGGCGCGGTTATTTTTTCATGTC 3′
6527	*E2: pagP*	Reverse 5′ GAAGGAATAGCCAGCCATGG 3′
6102	*F1: slyB*	Forward 5′ GGCCCGATTTCATACCTCTTCC 3′
6103	*F2: slyB*	Reverse 5′ ACCCCATCAGTGAAACGGCCAG 3′
9206	*G1: pdgL*	Forward 5′ CTGTACTACTGATTATCAAATG 3′
9207	*G2: pdgL*	Reverse 5′ CAGGCTGAACAGGAGGAAAG 3′
7981	*H1: pagD*	Forward 5′ CCCGCTCCGATTTAATCT 3′
7982	*H2: pagD*	Reverse 5′ CTGGCCAAAACATGGAATG 3′
8201	*I1: virK*	Forward 5′ CGCTTGCCGCCTTCCTGCAACT 3′
8202	*I2: virK*	Reverse 5′ CGATAAGGCGTCTGCGCCATG 3′
9297	*J1: ompX*	Forward 5′ GTCAGTACGAGTAAAGGTGGCA 3′
9298	*J2: ompX*	Reverse 5′ CCAGTGCTGAAAGACATGCA 3′
8203	*K1: pipD*	Forward 5′ CGCTATGCCGATGAACATAATATC 3′
8204	*K2: pipD*	Reverse 5′ GCCATCTTCGTTGCGCGCGA 3′
8431	*L1: ybjX*	Forward 5′ ATCATTCGCCAGCCAGCCATC 3′
8432	*L2: ybjX*	Reverse 5′ CGAAAGCTGGCTTTACGCCAG 3′
6891	*M1: mgtC*	Forward 5′ AATTCATGCAGGAGTAATATG 3′
1250	*M2: mgtC*	Reverse 5′ GAATTCTGGAAGAATAAGTACGTGC 3′
9732	*N1: rstA*	Forward 5′ AGAGGAATTAATCCGGCGA 3′
9733	*N2: rstA*	Reverse 5′ ACGCGGCTCAACAATGACATC 3′

Table S2. Promoter-specific primers used in SI nuclease-protection assay

Number	Name	Sequence
4518	*C3: yrbL*	Forward 5′ GATGACAGTCACCCCAAAACC 3′
4519	*C2: yrbL*	Reverse 5′ GGTCACGGTTGTAAATGAC 3′
9198	*J3: ompX*	Forward 5′ CGGCGGTTGAGGGTTCGTTGA 3′
9202	*J4: ompX*	Reverse 5′ CCAGAACAGCGGCCAGTGCTG 3′
8203	*K1: pipD*	Forward 5′ CCGCTATGCCGATGAACATAATATC 3′
9575	*K3: pipD*	Reverse 5′ GCGATGGGATGAATAACCTTATG 3′
6005	*M3: mgtC*	Forward 5′ GGAATTTATTGTTTAATGATTTCAGACG 3′
6002	*M4: mgtC*	Reverse 5′ CGCGTGTTGTAACATTATTGGGATG 3′

Table S3. Bacterial strains and plasmids used in this study

Strains	Description	Reference
S. enterica		
14028s	wild-type	ATCC
EG13918	*phoP*-HA	(Shin and Groisman, 2005)
DH5α	F⁻ *sup*E44 Δ*lac*U169 (ϕ80 *lac*ZΔM15) *hsd*R17 *rec*A1 *end*A1 *gyr*A96 *thi*-1 *rel*A1	(Hanahan, 1983)
Plasmids		
pMS201	rep_{pSC101} Km^R, promoterless *gfp* vector	(Mangan and Alon, 2003)
pMS-*virK*	rep_{pSC101} Km^R, P*virK gfp*	This work
pMS-*virK*-mut-up-for	rep_{pSC101} Km^R, P*virK*-mut-up-for *gfp*	This work
pMS-*virK*-mut-down	rep_{pSC101} Km^R, P*virK*-mut-down *gfp*	This work
pMS-virK-mut-up-rev	rep_{pSC101} Km^R, P*virK*-mut-down *gfp*	This work
pMS-*phoP4*	rep_{pSC101} Km^R, P*virK*-mut-up-rev *gfp*	This work
pMS-*phoP4*-rev	rep_{pSC101} Km^R, P*phoP4*-rev *gfp*	This work
pMS-*rstA4*	rep_{pSC101} Km^R, P*rstA4 gfp*	This work
pMS-*rstA4*-rev	rep_{pSC101} Km^R, P*rstA4*-rev *gfp*	This work
pMS-*phoP4*-rev-K1	rep_{pSC101} Km^R, P*phoP4*-rev-K1 *gfp*	This work
pMS-*phoP4*-rev-C	rep_{pSC101} Km^R, P*phoP4*-rev-C *gfp*	This work
pMS-*phoP*	rep_{pSC101} Km^R, P*phoP gfp*	This work
pMS-*rstA*	rep_{pSC101} Km^R, P*rstA gfp*	This work

pMS-*phoP4*-7nt	rep$_{pSC101}$ Km^R, P*phoP4*-7nt *gfp*	This work
pMS-*phoP4*-17nt	rep$_{pSC101}$ Km^R, P*phoP4*-17nt *gfp*	This work
pMS-*phoP4*-12nt	rep$_{pSC101}$ Km^R, P*phoP4*- 12nt *gfp*	This work
pMS-*phoP4*-21nt	rep$_{pSC101}$ Km^R, P*phoP4*+21nt *gfp*	This work
pMS-*phoP4*-22nt	rep$_{pSC101}$ Km^R, P*phoP4*-22nt *gfp*	This work
pMS-*phoP4*-23nt	rep$_{pSC101}$ Km^R, P*phoP4*-23nt *gfp*	This work
pMS-*phoP4*-24nt	rep$_{pSC101}$ Km^R, P*phoP4*-24nt *gfp*	This work
pMS-*phoP4*-25nt	rep$_{pSC101}$ Km^R, P*phoP4*-25nt *gfp*	This work
pMS-*phoP4*-26nt	rep$_{pSC101}$ Km^R, P*phoP4*-26nt *gfp*	This work
pMS-*phoP4*-27nt	rep$_{pSC101}$ Km^R, P*phoP4*- 27nt *gfp*	This work
pMS-*phoP4*-28nt	rep$_{pSC101}$ Km^R, P*phoP4*-28nt *gfp*	This work
pMS-*phoP4*-33nt	rep$_{pSC101}$ Km^R, P*phoP4*-33nt *gfp*	This work
pMS-*phoP4*-48nt	rep$_{pSC101}$ Km^R, P*phoP4*-48nt *gfp*	This work
pMS-*phoP4*-rev-28nt	rep$_{pSC101}$ Km^R, P*phoP4*-rev-28nt *gfp*	This work
pMS-*phoP4*-rev-29nt	rep$_{pSC101}$ Km^R, P*phoP4*-rev-29nt *gfp*	This work
pMS-*phoP4*-rev-30nt	rep$_{pSC101}$ Km^R, P*phoP4*-rev-30nt *gfp*	This work
pMS-*phoP4*-rev-31nt	rep$_{pSC101}$ Km^R, P*phoP4*-rev-31nt *gfp*	This work
pMS-*phoP4*-rev-36nt	rep$_{pSC101}$ Km^R, P*phoP4*-rev-36nt *gfp*	This work
pMS-*phoP4*-rev-47nt	rep$_{pSC101}$ Km^R, P*phoP4*-rev-47nt *gfp*	This work
pMS-*phoP4*-rev-26nt	rep$_{pSC101}$ Km^R, P*phoP4*-rev-26nt *gfp*	This work
pMS-*phoP4*-rev-27nt	rep$_{pSC101}$ Km^R, P*phoP4*-rev-27nt *gfp*	This work
pMS-*phoP4*-rev-32nt	rep$_{pSC101}$ Km^R, P*phoP4*-rev-32nt *gfp*	This work
pMS-*phoP4*-rev-37nt	rep$_{pSC101}$ Km^R, P*phoP4*-rev-37nt *gfp*	This work
pMS-*phoP4*-rev-48nt	rep$_{pSC101}$ Km^R, P*phoP4*-rev-48nt *gfp*	This work

pMS-*ybjX*	rep_{pSC101} Km^R, P*ybjX* *gfp*	This work
pMS-*ybjX*-mut-up	rep_{pSC101} Km^R, P*ybjX*-mut-up *gfp*	This work
pMS-*ybjX*-mut-down	rep_{pSC101} Km^R, P*ybjX*-mut-down *gfp*	This work
pMS-*virK-phoP4*	rep_{pSC101} Km^R, P*virK-phoP4* *gfp*	This work
pMS-*phoP4*-rev-23nt	rep_{pSC101} Km^R, P*phoP4*-rev-23nt *gfp*	This work
pMS-*phoP4*-rev-K1-TTTAAT	rep_{pSC101} Km^R, P*phoP4*-rev-K1-TTTAAT *gfp*	This work
pMS-*phoP4*-rev-C-TATAAT	rep_{pSC101} Km^R, P*phoP4*-rev-C-TATAAT *gfp*	This work
pMS-*phoP4*-rev-K1-TATAAT	rep_{pSC101} Km^R, P*phoP4*-rev-K1-TATAAT *gfp*	This work
pMS-*phoP4*-rev-K1-TTGACA	rep_{pSC101} Km^R, P*phoP4*-rev-K1-TTGACA *gfp*	This work
pMS-*phoP4*+11nt-TATGTT	rep_{pSC101} Km^R, P*phoP4*+11nt-TATGTT *gfp*	This work

Table S4. GFP primers used in this study

Number	Name	Sequence
9224	*virK*-F	Forward 5′ TCGA<br>TTTATTACCGCCATTGATAAACTGTTTAACAACATCGTCTGTACAGACCTTCTTCGTTGCCTTTACGTTTAACTCAATCAGGCTACC<br>GTCTCGGTTATAAGT 3′
9225	*virK*-R	Reverse 5′ GATC<br>ACTTATAACCGAGACGGTAGCCTGATTGAGTTAAACGTAAAGGCAACGAAGAAGGTCTGTACAGACGATGTTGTTAAACAGTTTATC<br>AATGGCGGTAATAAA 3′
9228	*virK*-mut-for-F (up)	Forward 5′ TCGA<br>TTTATTACCGCCATTGATAAACTGAATAACAACATCGTCTGTACAGACCTTCTTCGTTGCCTTTACGTTTAACTCAATCAGGCTACC<br>GTCTCGGTTATAAGT 3′
9229	*virK*-mut-for-R (up)	Reverse 5′ GATC<br>ACTTATAACCGAGACGGTAGCCTGATTGAGTTAAACGTAAAGGCAACGAAGAAGGTCTGTACAGACGATGTTGTTATTCAGTTTATC<br>AATGGCGGTAATAAA 3′
9230	*virK*-mut-down-F	Forward 5′ TCGA<br>TTTATTACCGCCATTGATAAACTGTTTAACAACATCGTCTGTACAGACCTTCTTCGTTGCCTTTACGAATAACTCAATCAGGCTACC<br>GTCTCGGTTATAAGT 3′
9231	*virK*-mut-down-R	Reverse 5′ GATC<br>ACTTATAACCGAGACGGTAGCCTGATTGAGTTATTCGTAAAGGCAACGAAGAAGGTCTGTACAGACGATGTTGTTAAACAGTTTATC<br>AATGGCGGTAATAAA 3′
9226	*virK*-mut-rev-F (up)	Forward 5′ TCGA<br>TTTATTACCGCCATTgattatctgtttaacaacaTCGTCTGTACAGACCTTCTTCGTTGCCTTTACGTTTAACTCAATCAGGCTACC<br>GTCTCGGTTATAAGT 3′
9227	*virK*-mut-rev-R (up)	Reverse 5′ GATC<br>ACTTATAACCGAGACGGTAGCCTGATTGAGTTAAACGTAAAGGCAACGAAGAAGGTCTGTACAGACGAtgttgttaaacagataatc<br>AATGGCGGTAATAAA 3′
8302	GFP-seq-F	Forward 5′ CCAGTTTACTTTGCAGGGCTTC 3′
7817	GFP-seq-R	Reverse 5′ GCCCATTAACATCACCATC 3′
8142	*phoP4*-F	Forward 5′ TCGA CTATTTGTCTGGTTTATTAACTGTTTATCCCCAAAGCACCATAATCAACGCTAGACTG 3′
8143	*phoP4*-R	Reverse 5′ GATC CAGTCTAGCGTTGATTATGGTGCTTTGGGGATAAACAGTTAATAAACCAGACAAATAG 3′
8233	*phoP4*-rev-F	Forward 5′ TCGACTATTTGTCATAAACAGTTAATAAACCACCCCAAAGCACCATAATCAACGCTAGACTG 3′

8234	*phoP4*-rev-R	Reverse 5′ GATC CAGTCTAGCGTTGATTATGGTGCTTTGGGGTGGTTTATTAACTGTTTATGACAAATAG 3′
8144	*rstA4*-F	Forward 5′ TCGA AACCGCTCTCGTTTAGAAAAGATTTATGGAAGGGAAAACAGAGGCGGTGTATGTTGGCGTTTTCTA 3′
8145	*rstA4*-R	Reverse 5′ GATC TAGAAAACGCCAACATACACCGCCTCTGTTTTCCCTTCCATAAATCTTTTCTAAACGAGAGCGGTT 3′
8235	*rstA4*-rev-F	Forward 5′ TCGA AACCGCTCATAAATCTTTTCTAAACGAGGAAGGGAAAACAGAGGCGGTGTATGTTGGCGTTTTCTA 3′
8236	*rstA4*-rev-R	Reverse 5′ GATC TAGAAAACGCCAACATACACCGCCTCTGTTTTCCCTTCCTCGTTTAGAAAAGATTTATGAGCGGTT 3′
8604	*phoP4*-rev-K1-F	Forward 5′ TCGA CTATTTGTCATAAACAGTTAATAAACCATTTTTATTTACTCACCTGATGGTAACCCCAAAGCACCATA ATCAACGCTAGACTG 3′
8592	*phoP4*-rev-K1-R	Reverse 5′ GATC CAGTCTAGCGTTGATTATGGTGCTTTGGGGTTACCATCAGGTGAGTAAATAAAAATGGTTTATTAA CTGTTTATGACAAATAG 3′
8489	*phoP4*-rev-C-F	Forward 5′ TCGA CTATTTGTCATAAACAGTTAATAAACCAAACGTCACTAAACCCGCCTTTGCACTTTACGGTTATCC CCAAAGCACCATAATCAACGCTAGACTG 3′
8490	*phoP4*-rev-C-R	Reverse 5′ GATC CAGTCTAGCGTTGATTATGGTGCTTTGGGGATAACCGTAAAGTGCAAAGGCGGGTTTAGTGACGTT TGGTTTATTAACTGTTTATGACAAATAG 3′
4811	*phoP*-F	Forward 5′ CGGCTCGAG AAGAGTTGACCCGTGGCAAGCGTG 3′
4432	*phoP*-R	Reverse 5′ CGGGATCC TGGCGTAATAATGCATTATCCTC 3′
4842	*rstA*-F	Forward 5′ CGGCTCGAG AAATAAGGAATGATCGACCAC 3′
5231	*rstA*-R	Reverse 5′ CGGGATCC CAGATAAGCGGCAATGAGAG 3′
8694	*phoP4*-7nt-F	Forward 5′ TCGA CTATTTGTCCCCCATGGTTTATTAACTGTTTATAAGCACCATAATCAACGCTAGACTG 3′
8693	*phoP4*-7nt-R	Reverse 5′ GATC CAGTCTAGCGTTGATTATGGTGCTTATAAACAGTTAATAAACCATGGGGGACAAATAG 3′
8142	*phoP4*-12nt-F	Forward 5′ TCGA CTATTTGTCTGGTTTATTAACTGTTTATCCCCAAAGCACCATAATCAACGCTAGACTG 3′
8143	*phoP4*-12nt-R	Reverse 5′ GATC CAGTCTAGCGTTGATTATGGTGCTTTGGGGATAAACAGTTAATAAACCAGACAAATAG 3′
8691	*phoP4*-17nt-F	Forward 5′ TCGA CTATTTGTCGGAAGGTGGTTTATTAACTGTTTATGAAAACCCCAAAGCACCATAATCAACGCTAGACTG 3′
8692	*phoP4*-17nt-R	Reverse 5′ GATC CAGTCTAGCGTTGATTATGGTGCTTTGGGGTTTTCATAAACAGTTAATAAACCACCTTCCGACAAATAG 3′
8736	*phoP4*-21nt-F	Forward 5′ TCGA CTATTTGTCTGGTTTATTAACTGTTTATGGAAGGGAACCCCAAAGCACCATAATCAACGCTAGACTG 3′
8749	*phoP4*-21nt-R	Reverse 5′ GATC CAGTCTAGCGTTGATTATGGTGCTTTGGGGTTCCCTTCCATAAACAGTTAATAAACCAGACAAATAG 3′
8737	*phoP4*-22nt-F	Forward 5′ TCGA CTATTTGTCTGGTTTATTAACTGTTTATGAAGGGAAAACCCCAAAGCACCATAATCAACGCTAGACTG 3′
8750	*phoP4*-22nt-R	Reverse 5′ GATC CAGTCTAGCGTTGATTATGGTGCTTTGGGGTTTTCCCTTCATAAACAGTTAATAAACCAGACAAATAG 3′
8545	*phoP4*-23nt-F	Forward 5′ TCGA CTATTTGTCTGGTTTATTAACTGTTTATGGAAGGGAAAACCCCAAAGCACCATAATCAACGCTAGACTG 3′

8548	*phoP4*-23nt-R	Reverse 5′ GATC CAGTCTAGCGTTGATTATGGTGCTTTGGGGTTTTCCCTTCCATAAACAGTTAATAAACCAGACAAATAG 3′
8738	*phoP4*-24nt-F	Forward 5′ TCGA CTATTTGTCTGGTTTATTAACTGTTTATTGGAAGGGAAAACCCCAAAGCACCATAATCAACGCTAGACTG 3′
8751	*phoP4*-24nt-R	Reverse 5′ GATC CAGTCTAGCGTTGATTATGGTGCTTTGGGGTTTTCCCTTCCAATAAACAGTTAATAAACCAGACAAATAG 3′
8739	*phoP4*-25nt-F	Forward 5′ TCGA CTATTTGTCTGGTTTATTAACTGTTTATCTGGAAGGGAAAACCCCAAAGCACCATAATCAACGCTAGACTG 3′
8752	*phoP4*-25nt-R	Reverse 5′ GATC CAGTCTAGCGTTGATTATGGTGCTTTGGGGTTTTCCCTTCCAGATAAACAGTTAATAAACCAGACAAATAG 3′
8740	*phoP4*-26nt-F	Forward 5′ TCGA CTATTTGTCTGGTTTATTAACTGTTTATTCTGGAAGGGAAAACCCCAAAGCACCATAATCAACGCTAGACTG 3′
8753	*phoP4*-26nt-R	Reverse 5′ GATC CAGTCTAGCGTTGATTATGGTGCTTTGGGGTTTTCCCTTCCAGAATAAACAGTTAATAAACCAGACAAATAG 3′
8741	*phoP4*-27nt-F	Forward 5′ TCGA CTATTTGTCTGGTTTATTAACTGTTTATCTCTGGAAGGGAAAACCCCAAAGCACCATAATCAACGCT AGACTG 3′
8754	*phoP4*-27nt-R	Reverse 5′ GATC CAGTCTAGCGTTGATTATGGTGCTTTGGGGTTTTCCCTTCCAGAGATAAACAGTTAATAAACCAGACAAATAG 3′
8613	*phoP4*-28nt-F	Forward 5′ TCGA CTATTTGTCTGGTTTATTAACTGTTTATGCTCTGGAAGGGAAAACCCCAAAGCACCATAATCAACGCTAGACTG 3′
8593	*phoP4*-28nt-R	Reverse 5′ GATC CAGTCTAGCGTTGATTATGGTGCTTTGGGGTTTTCCCTTCCAGAGCATAAACAGTTAATAAACCAGACAAATAG 3′
8597	*phoP4*-33nt-F	Forward 5′ TCGA CTATTTGTCTGGTTTATTAACTGTTTATGAACCGCTCTGGAAGGGAAAACCCCAAAGCACCATAATCAAC GCTAGACTG 3′
8602	*phoP4*-33nt-R	Reverse 5′ GATC CAGTCTAGCGTTGATTATGGTGCTTTGGGGTTTTCCCTTCCAGAGCGGTTCATAAACAGTTAATAAACC AGACAAATAG 3′
8487	*phoP4*-48nt-F	Forward 5′ TCGA CTATTTGTCTGGTTTATTAACTGTTTATAACGTCACTAAACCCGCCTTTGCACTTTACGGTTATCCCCAAAGCACCATAATCAACGC TAGACTG 3′

8488	*phoP4*-48nt-R	Reverse 5′ GATC CAGTCTAGCGTTGATTATGGTGCTTTGGGGATAACCGTAAAGTGCAAAGGCGGGTTTAGTGACGTTATAAACAGTTAATAAACCAGA CAAATAG 3′
8733	*phoP4*-rev-26nt-F	Forward 5′ TCGA CTATTTGTCATAAACAGTTAATAAACCATCACCTGATGGTAACCCCAAAGCACCATAATCAACGCTAGACTG 3′
8746	*phoP4*-rev-26nt-R	Reverse 5′ GATC CAGTCTAGCGTTGATTATGGTGCTTTGGGGTTACCATCAGGTGATGGTTTATTAACTGTTTATGACAAATAG 3′
8732	*phoP4*-rev-28nt-F	Forward 5′ TCGA CTATTTGTCATAAACAGTTAATAAACCAACTCACCTGATGGTAACCCCAAAGCACCATAATCAACGCTAGACTG 3′
8745	*phoP4*-rev-28nt-R	Reverse 5′ GATC CAGTCTAGCGTTGATTATGGTGCTTTGGGGTTACCATCAGGTGAGTTGGTTTATTAACTGTTTATGACAAATAG 3′
8731	*phoP4*-rev-29nt-F	Forward 5′ TCGA CTATTTGTCATAAACAGTTAATAAACCATACTCACCTGATGGTAACCCCAAAGCACCATAATCAACGCTAGACTG 3′
8744	*phoP4*-rev-29nt-R	Reverse 5′ GATC CAGTCTAGCGTTGATTATGGTGCTTTGGGGTTACCATCAGGTGAGTATGGTTTATTAACTGTTTATGACAAATAG 3′
8730	*phoP4*-rev-30nt-F	Forward 5′ TCGA CTATTTGTCATAAACAGTTAATAAACCATTACTCACCTGATGGTAACCCCAAAGCACCATAATCAACGCTAGACTG 3′
8743	*phoP4*-rev-30nt-R	Reverse 5′ GATC CAGTCTAGCGTTGATTATGGTGCTTTGGGGTTACCATCAGGTGAGTAATGGTTTATTAACTGTTTATGACAAATAG 3′
8729	*phoP4*-rev-31nt-F	Forward 5′ TCGA CTATTTGTCATAAACAGTTAATAAACCATTTACTCACCTGATGGTAACCCCAAAGCACCATAATCAACGCTAGACTG 3′
8742	*phoP4*-rev-31nt-R	Reverse 5′ GATC CAGTCTAGCGTTGATTATGGTGCTTTGGGGTTACCATCAGGTGAGTAAATGGTTTATTAACTGTTTATGACAAATAG 3′
8441	*phoP4*-rev-36nt-F	Forward 5′ TCGA CTATTTGTCATAAACAGTTAATAAACCATTTTATTTACTCACCTGATGGTAACCCCAAAGCACCATAATCAACGCTAGACTG 3′
8438	*phoP4*-rev-36nt-R	Reverse 5′ GATC CAGTCTAGCGTTGATTATGGTGCTTTGGGGTTACCATCAGGTGAGTAAATAAAATGGTTTATTAACTGTTTATGACAAATAG 3′
8604	*phoP4*-rev-37-F	Forward 5′ TCGA CTATTTGTCATAAACAGTTAATAAACCATTTTTATTTACTCACCTGATGGTAACCCCAAAGCACCATAATCAACGCTAGACTG 3′
8592	*phoP4*-rev-37-R	Reverse 5′ GATC CAGTCTAGCGTTGATTATGGTGCTTTGGGGTTACCATCAGGTGAGTAAATAAAAATGGTTTATTAACTGTTTATGACAAATAG 3′

8734	*phoP4*-rev-47nt-F	Forward 5′ TCGA CTATTTGTCATAAACAGTTAATAAACCATTATACTTGCTTTTTATTTACTCACCTGATGGTAACCCCAAAGCACCATA ATCAACGCTAGACTG 3′
8747	*phoP4*-rev-47nt-R	Reverse 5′ GATCCAGTCTAGCGTTGATTATGGTGCTTTGGGGTTACCATCAGGTGAGTAAATAAAAAGCAAGTATAATGGTTTATTA ACTGTTTATGACAAATAG 3′
8608	*phoP4*-rev-27nt-F	Forward 5′ TCGAC TATTTGTCATAAACAGTTAATAAACCACTCACCTGATGGTAACCCCAAAGCACCATAATCAACGCTAGACTG 3′
8601	*phoP4*-rev-27nt-R	Reverse 5′ GATC CAGTCTAGCGTTGATTATGGTGCTTTGGGGTTACCATCAGGTGAGTGGTTTATTAACTGTTTATGACAAATAG 3′
8598	*phoP4*-rev-32nt-F	Forward 5′ TCGA CTATTTGTCATAAACAGTTAATAAACCAATTTACTCACCTGATGGTAACCCCAAAGCACCATAATCAACGCTAGACTG 3′
8606	*phoP4*-rev-32nt-R	Reverse 5′ GATC CAGTCTAGCGTTGATTATGGTGCTTTGGGGTTACCATCAGGTGAGTAAATTGGTTTATTAACTGTTTATGACAAATAG 3′
8489	*phoP4*-rev-48nt-F	Forward 5′ TCGA CTATTTGTCATAAACAGTTAATAAACCAAACGTCACTAAACCCGCCTTTGCACTTTACGGTTATCCCCAAAGCACCA TAATCAACGCTAGACTG 3′
8490	*phoP4*-rev-48nt-R	Reverse 5′ GATC CAGTCTAGCGTTGATTATGGTGCTTTGGGGATAACCGTAAAGTGCAAAGGCGGGTTTAGTGACGTTTGGTTTATTAAC TGTTTATGACAAATAG 3′
9253	*ybjX4*	Forward 5′ TCGA GCAAAATTTTAACCAGAAAGTAAACCGTATCTAAACAAACCTGACGCAATAAAGATAGCGCCGGCTGTATTGACGATTGGTTAATGT TTCCTGTGGTTCAATCTCGACAATCT 3′
9255	*ybjX4*	Reverse 5′ GATC AGATTGTCGAGATTGAACCACAGGAAACATTAACCAATCGTCAATACAGCCGGCGCTATCTTTATTGCGTCAGGTTTGTTTAGATAC GGTTTACTTTCTGGTTAAAATTTTGC 3′
9272	*ybjX4*-mut-up	Forward 5′ TCGA GCAAAATTTTAACCAGAAAGTAAACCGTATCTTATGAAACCTGACGCAATAAAGATAGCGCCGGCTGTATTGACGATTGGTTAATGT TTCCTGTGGTTCAATCTCGACAATCT 3′
9273	*ybjX4*-mut-up	Reverse 5′ GATC AGATTGTCGAGATTGAACCACAGGAAACATTAACCAATCGTCAATACAGCCGGCGCTATCTTTATTGCGTCAGGTTTCATAAGATAC GGTTTACTTTCTGGTTAAAATTTTGC 3′
9293	*ybjX4*-mut-dn	Forward 5′ TCGA GCAAAATTTTAACCAGAAAGTAAACCGTATCTAAACAAACCTGACGCAATAAAGATAGCGCCGGCTGTATTGACGATTGGATATTGT TTCCTGTGGTTCAATCTCGACAATCT 3′
9295	*ybjX4*-mut-dn	Reverse 5′ GATC AGATTGTCGAGATTGAACCACAGGAAACAATATCCAATCGTCAATACAGCCGGCGCTATCTTTATTGCGTCAGGTTTGTTTAGATAC GGTTTACTTTCTGGTTAAAATTTTGC 3′

9436	*virK-phoP4*	Forward 5′ TCGA TTTATTACCGCCATTGATAAACTGTTTAACAACATCGTCTGTACAGACCTTCTCTGGTTTATTAACTGTTTATCCCCAAAGCACCATAATCAACGCTAGACTG 3′
9437	*virK-phoP4*	Reverse 5′ GATC CAGTCTAGCGTTGATTATGGTGCTTTGGGGATAAACAGTTAATAAACCAGAGAAGGTCTGTACAGACGATGTTGTTAAACAGTTTATCAATGGCGGTAATAAA 3′
8235	*phoP4*-rev-23nt-F	Forward 5′ TCGA AACCGCTCATAAATCTTTTCTAAACGAGGAAGGGAAAACAGAGGCGGTGTATGTTGGCGTTTTCTA 3′
8236	*phoP4*-rev-23nt-R	Reverse 5′ GATC TAGAAAACGCCAACATACACCGCCTCTGTTTTCCCTTCCTCGTTTAGAAAAGATTTATGAGCGGTT 3′
9183	*phoP4*-rev-K1-TTTAAT	Forward 5′ TCGA CTATTTGTCATAAACAGTTAATAAACCATTTTTATTTACTCACCTGATGGTAACCCCAAAGCACTTTAATCAACGCTAGACTG 3′
9184	*phoP4*-rev-K1-TTTAAT	Reverse 5′ GATC CAGTCTAGCGTTGATTAAAGTGCTTTGGGGTTACCATCAGGTGAGTAAATAAAAATGGTTTATTAACTGTTTATGACAAATAG 3′
9186	*phoP4*-rev-K1-TAATAT	Forward 5′ TCGA CTATTTGTCATAAACAGTTAATAAACCATTTTTATTTACTCACCTGATGGTAACCCCAAAGCACTAATATCAACGCTAGACTG 3′
9189	*phoP4*-rev-K1-TAATAT	Reverse 5′ GATC CAGTCTAGCGTTGATATTAGTGCTTTGGGGTTACCATCAGGTGAGTAAATAAAAATGGTTTATTAACTGTTTATGACAAATAG 3′
8524	*phoP4*-rev-C-*TATAAT*	Forward 5′ TCGA CTATTTGTCATAAACAGTTAATAAACCAAACGTCACTAAACCCGCCTTTGCACTTTACGGTTATCCCCAAAGCACTATAATCAACGCTAGACTG 3′
8521	*phoP4*-rev-C-*TATAAT*	Reverse 5′ GATC CAGTCTAGCGTTGATTATAGTGCTTTGGGGATAACCGTAAAGTGCAAAGGCGGGTTTAGTGACGTTTGGTTTATTAACTGTTTATGACAAATAG 3′
8610	*phoP4*-rev-K1-TTGACA	Forward 5′ TCGA CTATTTGTCATAAACAGTTAATAAACCATTTTTATTTACTCTTGACATGGTAACCCCAAAGCACCATAATCAACGCTAGACTG 3′
8607	*phoP4*-rev-K1-TTGACA	Reverse 5′ GATC CAGTCTAGCGTTGATTATGGTGCTTTGGGGTTACCATGTCAAGAGTAAATAAAAATGGTTTATTAACTGTTTATGACAAATAG 3′
9188	*phoP4*+11nt-TATGTT	Forward 5′ TCGA CTATTTGTCTGGTTTATTAACTGTTTATGGAAGGGAAAACCCCAAAGCACTATGTTCAACGCTAGACTG 3′
9181	*phoP4*+11nt-TATGTT	Reverse 5′ GATC CAGTCTAGCGTTGAACATAGTGCTTTGGGGTTTTCCCTTCCATAAACAGTTAATAAACCAGACAAATAG 3′

References

Hanahan, D. (1983) Studies on transformation of *Escherichia coli* with plasmids. *J Mol Biol* **166**: 557-580.

Mangan, S., and Alon, U. (2003) Structure and function of the feed-forward loop network motif. *Proc Natl Acad Sci USA* **100**: 11980-11985.

Shin, D., and Groisman, E.A. (2005) Signal-dependent binding of the response regulators PhoP and PmrA to their target promoters *in vivo*. *J Biol Chem* **280**: 4089-4094.

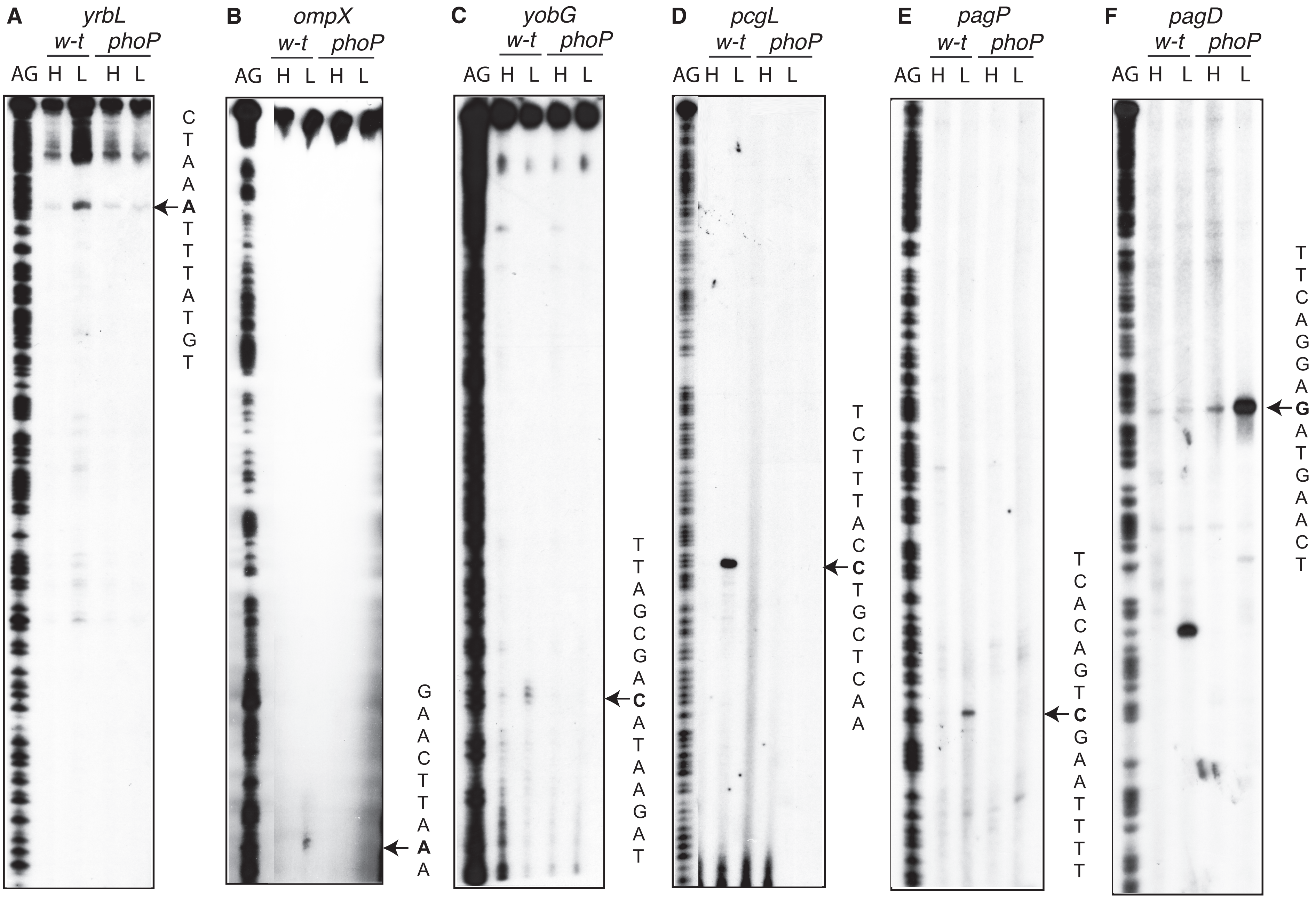

A yrbL w-t phoP AG H L H L
C T A A A T T T A T G T
B ompX w-t phoP AG H L H L
G A A C T T A A A
C yobG w-t phoP AG H L H L
T T A G C G A C A T A A G A T
D pcgL w-t phoP AG H L H L
T C T T T A C C T G C T C A A
E pagP w-t phoP AG H L H L
T C A C A G T C G A A T T T T
F pagD w-t phoP AG H L H L
T T C A G G A G A T G A A C T

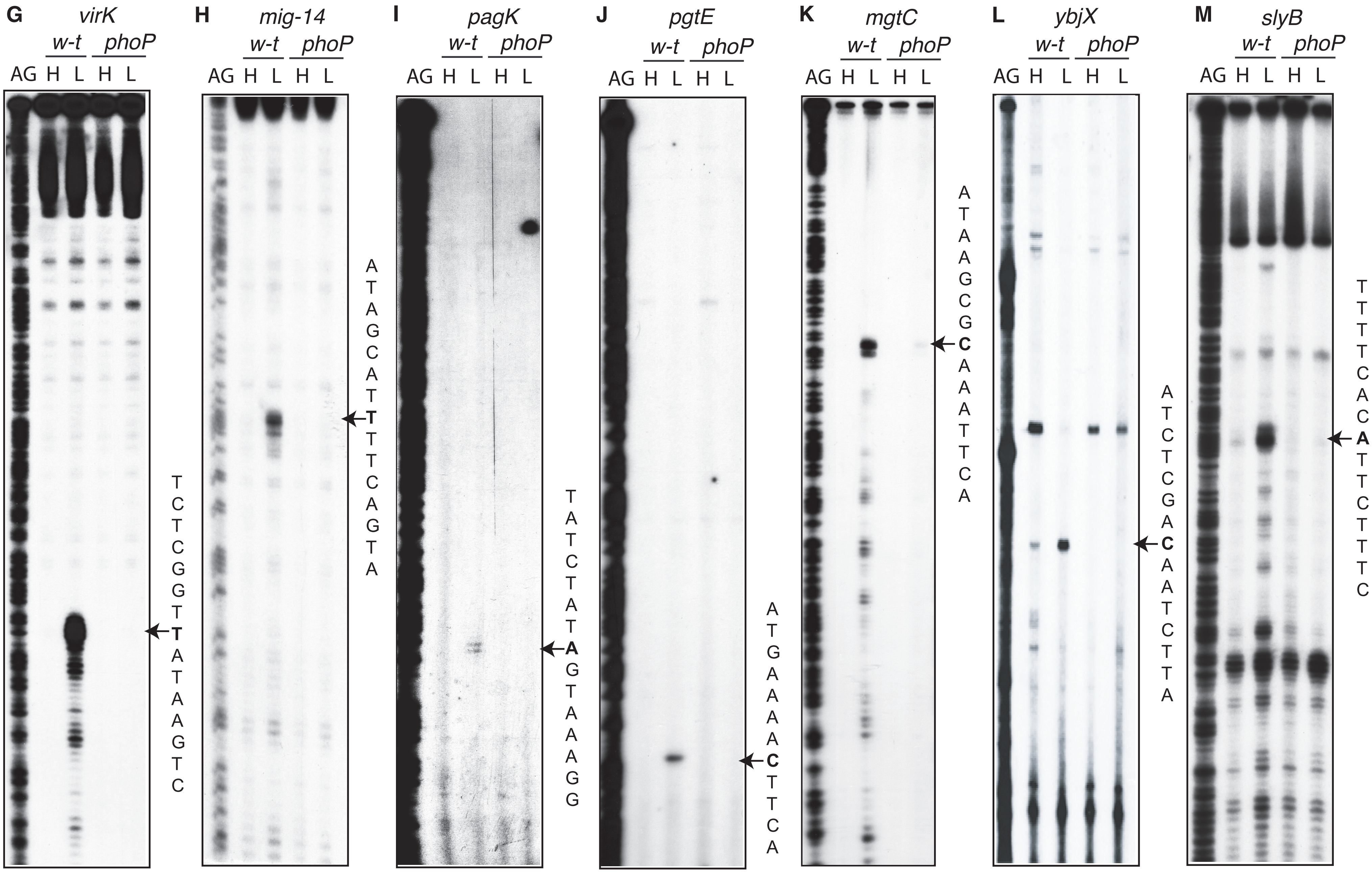
G virK
w-t phoP
AG H L H L
T C T C G G T T A T A A G T C
H mig-14
w-t phoP
AG H L H L
A T A G C A T T T T C A G T A
I pagK
w-t phoP
AG H L H L
T A T C T A T A G T A A A G
J pgtE
w-t phoP
AG H L H L
A T G A A A A C T T C A
K mgtC
w-t phoP
AG H L H L
A T A A G C G C A A A T T C A
L ybjX
w-t phoP
AG H L H L
A T C T C G A C A A T C T T A
M slyB
w-t phoP
AG H L H L
T T T T C A C A T T C T T T C

Figure S1

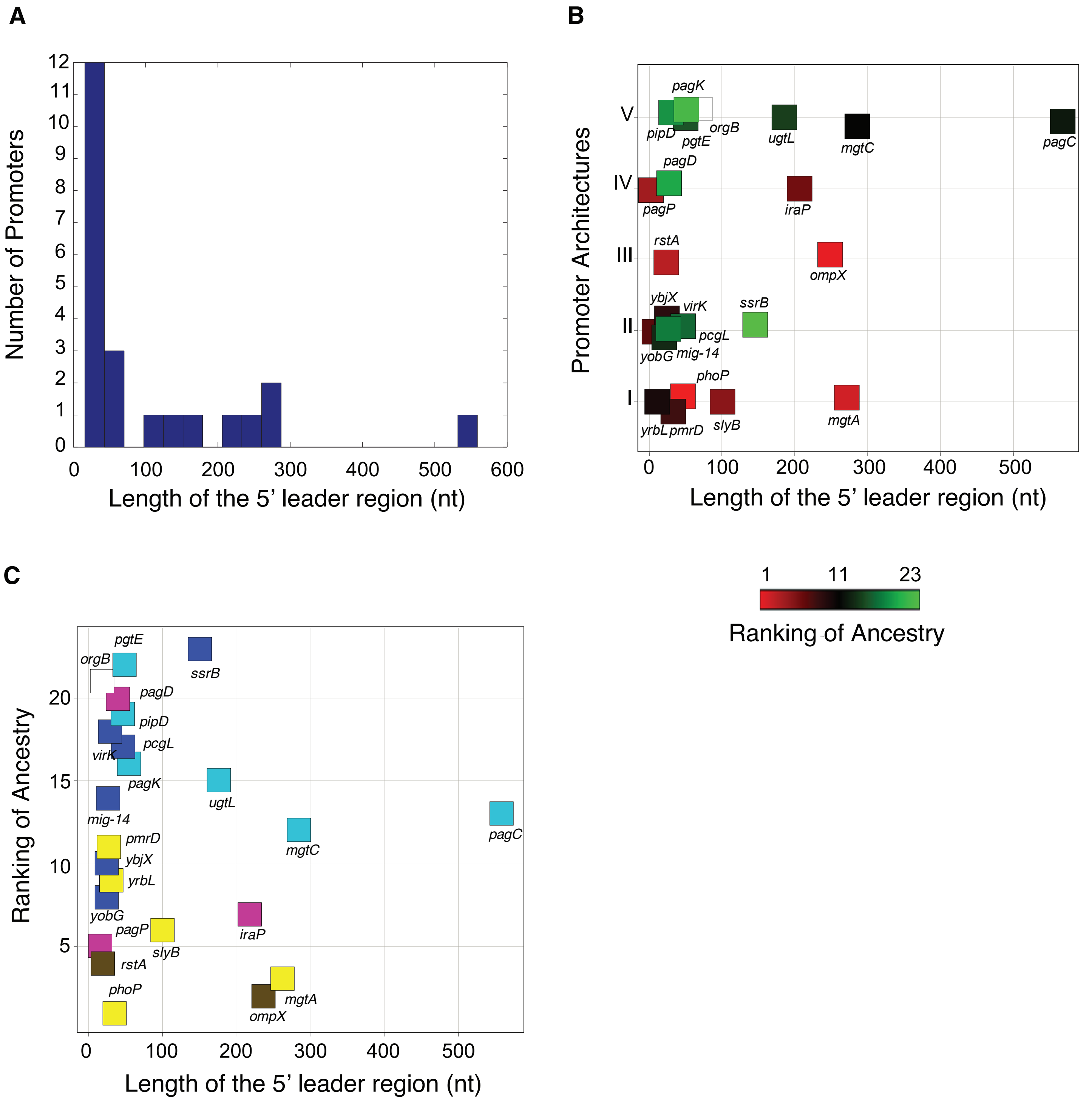

A
Number of Promoters
0
1
2
3
4
5
6
7
8
9
10
11
12
0
100
200
300
400
500
600
Length of the 5’ leader region (nt)
B
Promoter Architectures
V
IV
III
II
I
pagK
pipD
pgtE
orgB
ugtL
mgtC
pagC
pagD
pagP
iraP
rstA
ompX
ybjX
virK
ssrB
yobG
mig-14
pcgL
phoP
yrbL
pmrD
slyB
mgtA
0
100
200
300
400
500
Length of the 5’ leader region (nt)
C
Ranking of Ancestry
5
10
15
20
pgtE
orgB
ssrB
pagD
pipD
virK
pcgL
pagK
ugtL
mig-14
pagC
mgtC
pmrD
ybjX
yrbL
yobG
pagP
iraP
slyB
rstA
phoP
mgtA
ompX
0
100
200
300
400
500
Length of the 5’ leader region (nt)
1
11
23
Ranking of Ancestry

Figure S2

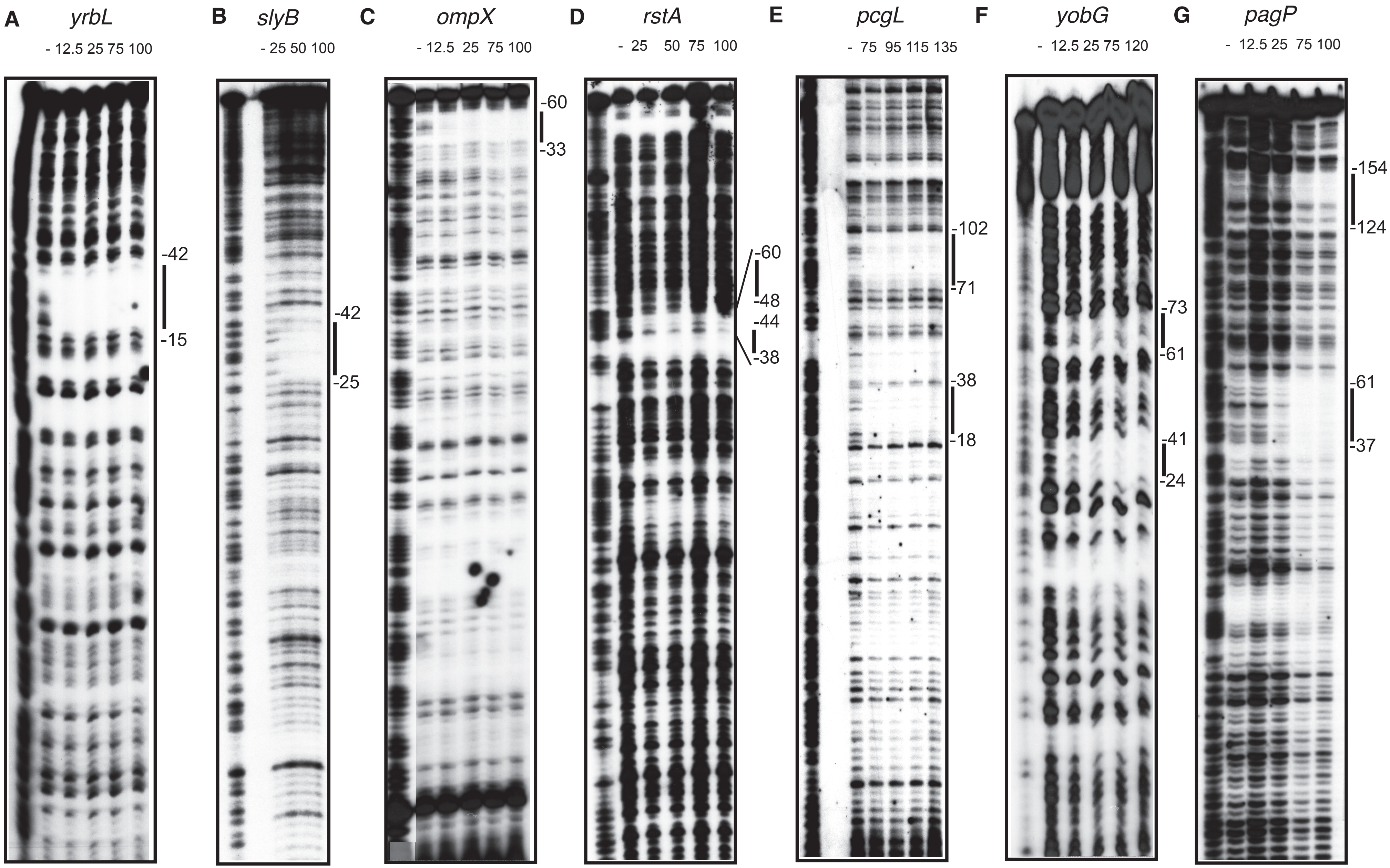
A
yrbL
- 12.5 25 75 100
-42
-15
B
slyB
- 25 50 100
-42
-25
C
ompX
- 12.5 25 75 100
-60
-33
D
rstA
- 25 50 75 100
-60
-48
-44
-38
E
pcgL
- 75 95 115 135
-102
-71
-38
-18
F
yobG
- 12.5 25 75 120
-73
-61
-41
-24
G
pagP
- 12.5 25 75 100
-154
-124
-61
-37

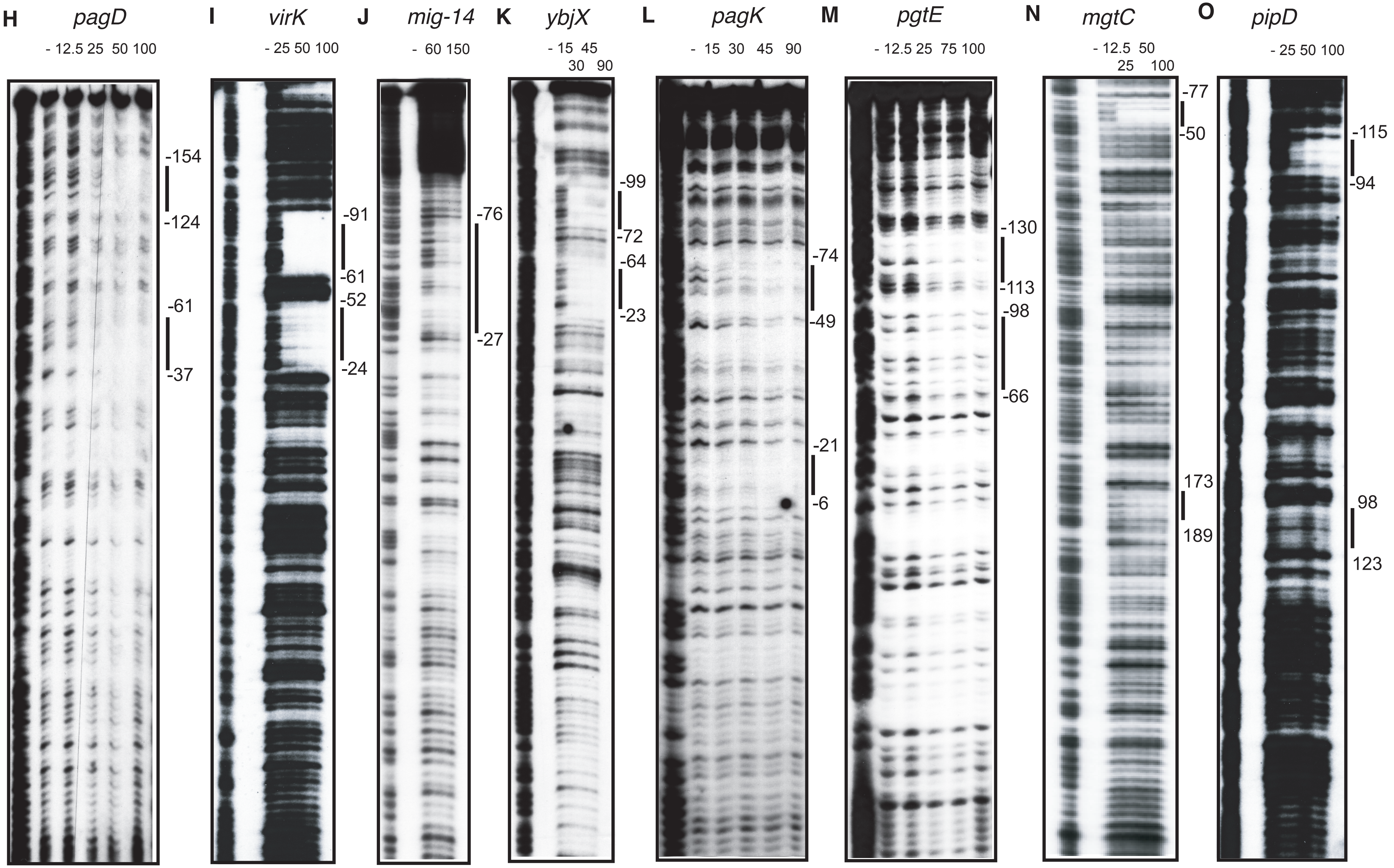

H pagD
- 12.5 25 50 100
-154
-124
-61
-37
I virK
- 25 50 100
-91
-61
-52
-24
J mig-14
- 60 150
-76
-27
K ybjX
- 15 45
30 90
-99
-72
-64
-23
L pagK
- 15 30 45 90
-74
-49
-21
-6
M pgtE
- 12.5 25 75 100
-130
-113
-98
-66
N mgtC
- 12.5 50
25 100
-77
-50
173
189
O pipD
- 25 50 100
-115
-94
98
123

Figure S3

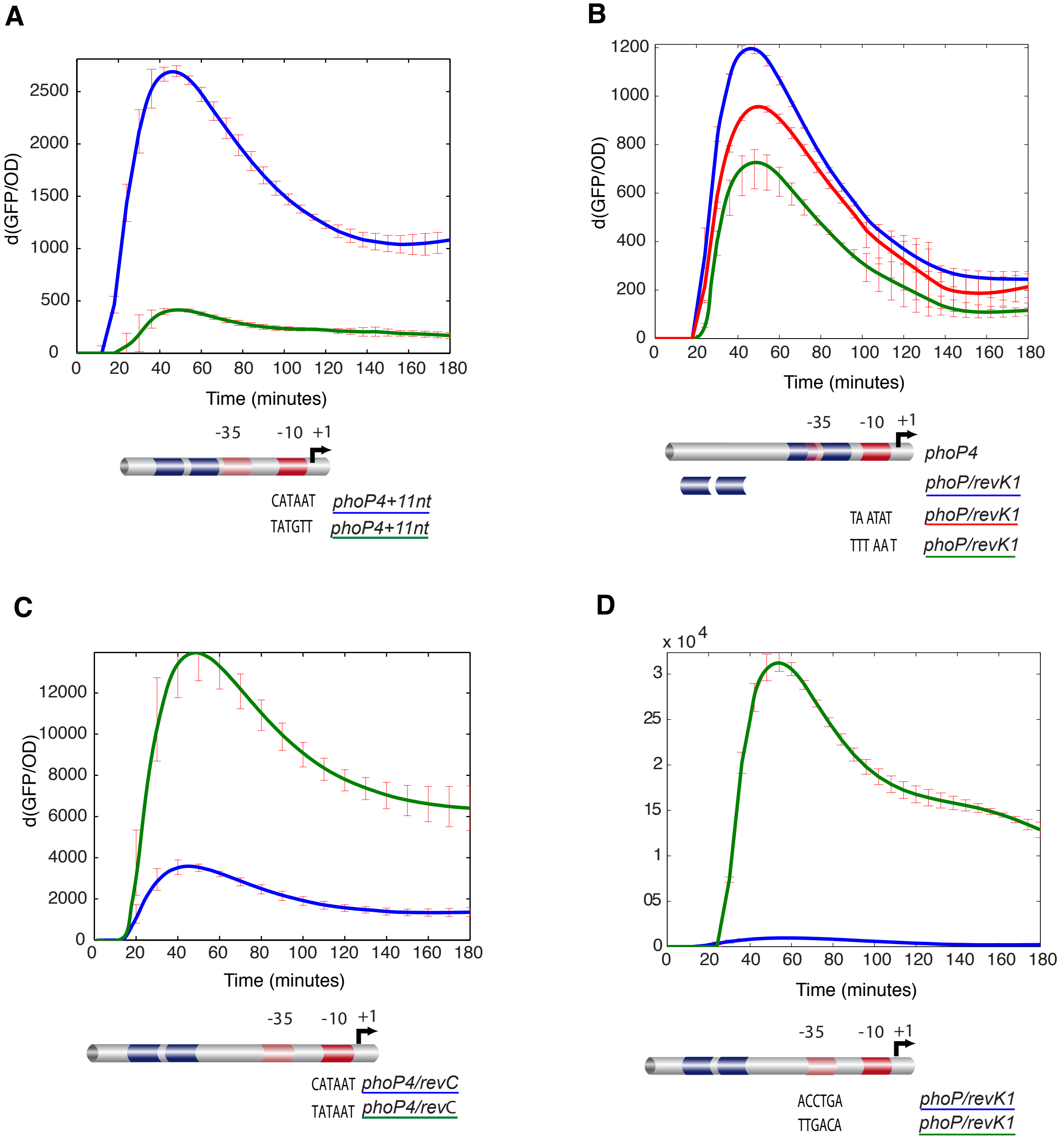
A
B
C
D
d(GFP/OD)
Time (minutes)
x 10^4
-35
-10
+1
CATAAT phoP4+11nt
TATGTT phoP4+11nt
phoP4
phoP/revK1
TA ATAT phoP/revK1
TTT AAT phoP/revK1
CATAAT phoP4/revC
TATAAT phoP4/revC
ACCTGA phoP/revK1
TTGACA phoP/revK1

Figure S4
